# Investigation of the role of stereoelectronic effects in the conformation of piperidones by NMR spectroscopy and X-ray diffraction

**DOI:** 10.3762/bjoc.11.213

**Published:** 2015-10-22

**Authors:** Cesar Garcias-Morales, David Ortegón-Reyna, Armando Ariza-Castolo

**Affiliations:** 1 Departamento de Química, Centro de Investigación y de Estudios Avanzados del Instituto Politécnico Nacional, Avenida Instituto Politécnico Nacional 2508 Colonia San Pedro Zacatenco, C.P. 07360, México, D.F., Mexico

**Keywords:** NMR spectroscopy, piperidone, spin–spin coupling constant, stereoelectronic effect, X-ray diffraction structure

## Abstract

This paper reports the synthesis of a series of piperidones **1**–**8** by the Mannich reaction and analysis of their structures and conformations in solution by NMR and mass spectrometry. The six-membered rings in 2,4,6,8-tetraphenyl-3,7-diazabicyclo[3.3.1]nonan-9-ones, compounds **1** and **2**, adopt a chair–boat conformation, while those in 2,4-diphenyl-3-azabicyclo[3.3.1]nonan-9-ones, compounds **3**–**8**, adopt a chair–chair conformation because of stereoelectronic effects. These stereoelectronic effects were analyzed by the ^1^*J*_C–H_ coupling constants, which were measured in the ^13^C satellites of the ^1^H NMR spectra obtained with the hetero-dqf pulse sequence. In the solid state, these stereoelectronic effects were investigated by measurement of X-ray diffraction data, the molecular geometry (torsional bond angles and bond distances), and inter- and intramolecular interactions, and by natural bond orbital analysis, which was performed using density functional theory at the ωB97XD/6311++G(d,p) level. We found that one of the main factors influencing the conformational stability of **3**–**8** is the interaction between the lone-pair electrons of nitrogen and the antibonding sigma orbital of C(7)–H_eq_ (n_N_→σ*_C–H(7)eq_), a type of hyperconjugative interaction.

## Introduction

Stereoelectronic effects have attracted the attention of many researchers with an interest in organic chemistry because of the major role that conformation plays in molecules and biomolecules; in addition, such effects are related to the spatial orientation of the orbital [1–3], which determines the stability of a structure and its reactivity [4–5]. One of the most important stereoelectronic effects is hyperconjugation, which is related to the anomeric effect (effect where a heteroatomic substituent adjacent to a heteroatom within a cyclohexane ring prefers the axial orientation instead of the equatorial) [6–12].

Moreover, stereoelectronic effects have been related to the stabilization of carbanions [13–14], carbocations [15–18], and free radicals [19–21] which has been explained by negative (n_X_→σ*_C–Y_) or positive hyperconjugation (σ_C–Y_→π* or p).

**Figure 1:**
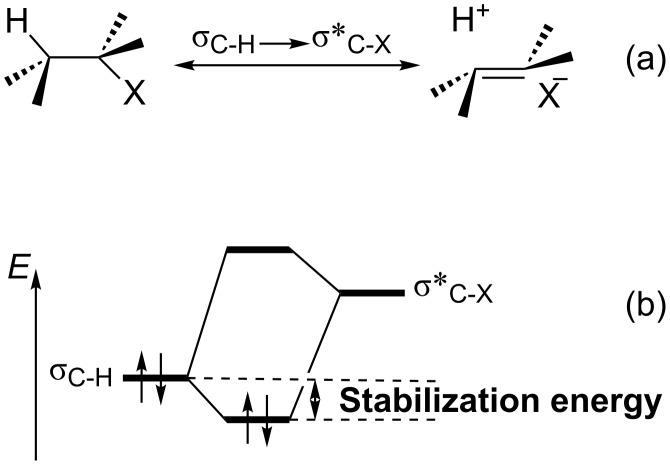
(a) Schematic representation of the vicinal σ_C−H_→σ*_C−X_ interaction by double-bond/no-bond resonance. (b) Stabilization energy because of the hyperconjugative interaction between σ_C−H_ and σ*_C−X_ orbitals.

Hyperconjugation is commonly described as the interaction between electronic orbitals where one filled orbital (donor) interacts with another unfilled orbital (acceptor) with the presence of an additional resonance structure (double-bond/no-bond resonance (Figure 1a). This interaction is also accompanied by stabilization of the molecule (Figure 1b) [1–3].

**Figure 2:**
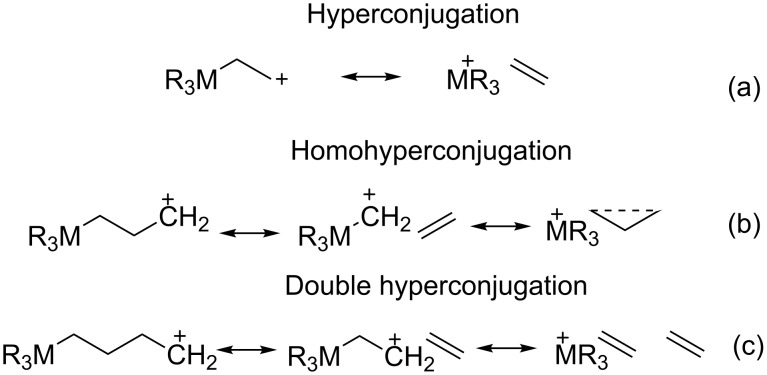
Schematic representation of stereoelectronic effects (a) hyperconjugation, (b) homohyperconjugation, (c) double hyperconjugation.

There is evidence that the interaction between electronic orbitals filled orbital (donor) with another unfilled orbital (acceptor) can be observed several bonds away from the orbitals. This effect is classified as hyperconjugation (electronic delocalization placing a σ-bridge between a donor and an acceptor orbital, Figure 2a), homohyperconjugation, considered the result of the direct through-space interactions between donor and acceptor orbitals is observed when a saturated center intervenes (the phenomenon is called γ-effect when the acceptor is a cationic p-orbital, Figure 2b), homoanomeric effect (when the acceptor is a σ*-orbital and the donor is a lone pair), and double hyperconjugation (extends the delocalization range even further by placing a σ-bridge between a donor and an acceptor, Figure 2c) [22–27].

**Figure 3:**
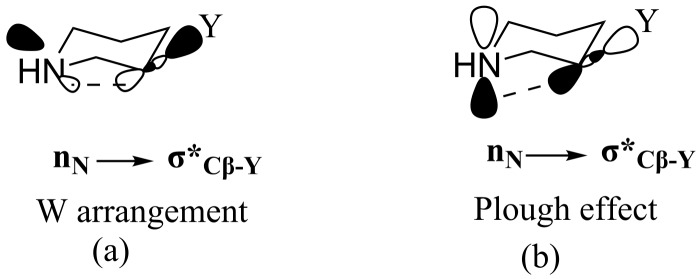
Schematic representation of possible homoanomeric interactions in six-membered saturated heterocycles. (a) Homohyperconjugation through the W arrangement, (b) homohyperconjugation through the Plough effect.

In the six-membered saturated heterocycles the more stable conformation is the one where the substituent on the β-carbon is equatorial with respect to the heteroatom, this is known as homoanomeric effect, which is a type of homohyperconjugation [28–36]. The homoanomeric effect can be observed in two cases: the first-one through a W-arrangement, where the lone-pair electrons (LPEs) of O, N, and S, on the pseudo equatorial position interact with the antibonding σ orbital (n_X_→σ*_Cβ–Y_) and the second-one is the Plough effect, where the lone-pair electrons (LPEs) of O, N, and S, on the pseudo axial position interact with the antibonding σ orbital (n_X_→σ*_Cβ–Y_) Figure 3 [22,37–44].

For the study of this kind of interaction, the NMR technique is a useful tool as it is a highly sensitive spectroscopical method, and NMR spin–spin coupling constants are used to experimentally investigate stereoelectronic effects [6–12,30–36,42–43]. In particular, it is acknowledged that delocalization interactions from electrons have a relatively important Fermi contact contribution [45]. For example in cyclohexane the spin–scalar coupling constant of the equatorial hydrogen (H_eq_) is 4 Hz higher (^1^*J*_C,Heq_) than the axial one (^1^*J*_C,Hax_). This difference in the ^1^*J*_C,H_ values has been explained in terms of delocalization interactions from electron hyperconjugation.

**Figure 4:**
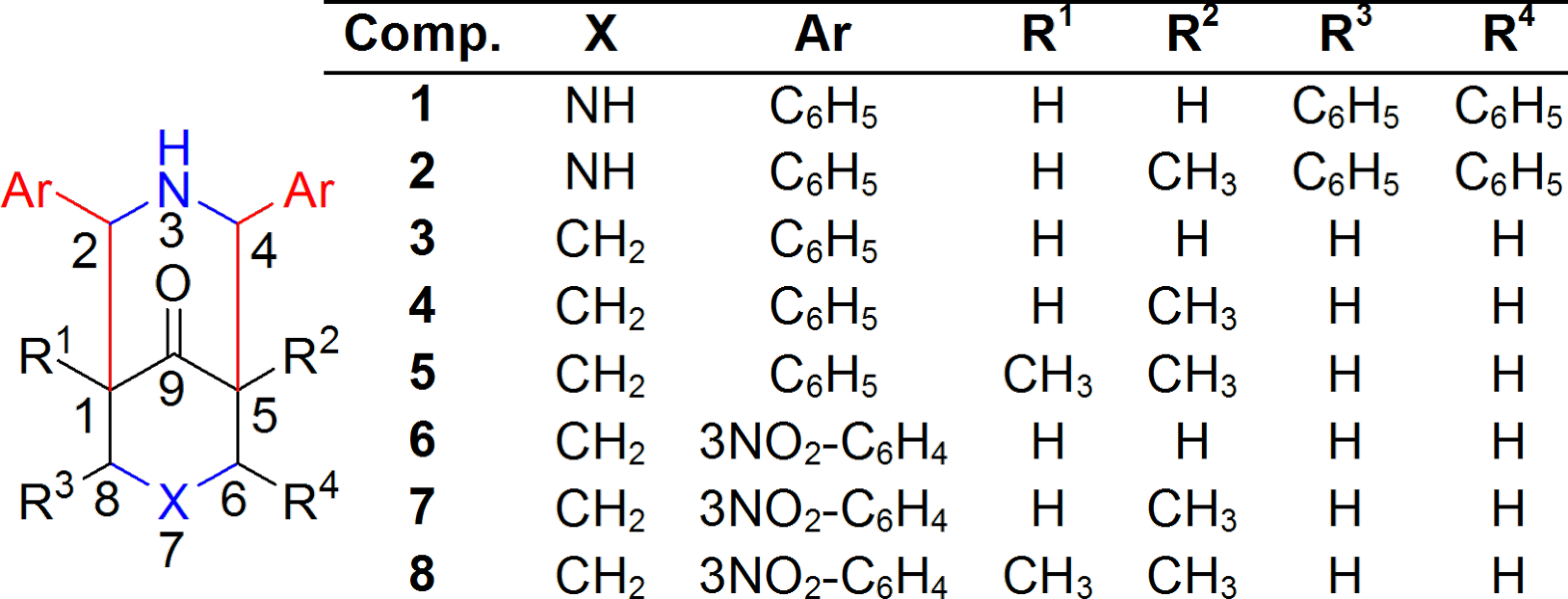
Structure of compounds **1** to **8**.

This study describes the synthesis of a series of 2,4-diphenyl-3-azabicyclo[3.3.1]nonan-9-one compounds (Figure 4) with restricted conformations. The complete analysis of the chemical shifts and the indirect coupling constants gives information about the electronic density and the interaction through space between the LPE of nitrogen with the antibonding σ_*C(7)–Heq_ orbital (n_N_→σ*c_(7)–Heq_), the results are supported by natural bond orbital (NBO) analysis.

## Results and Discussion

**Scheme 1:**
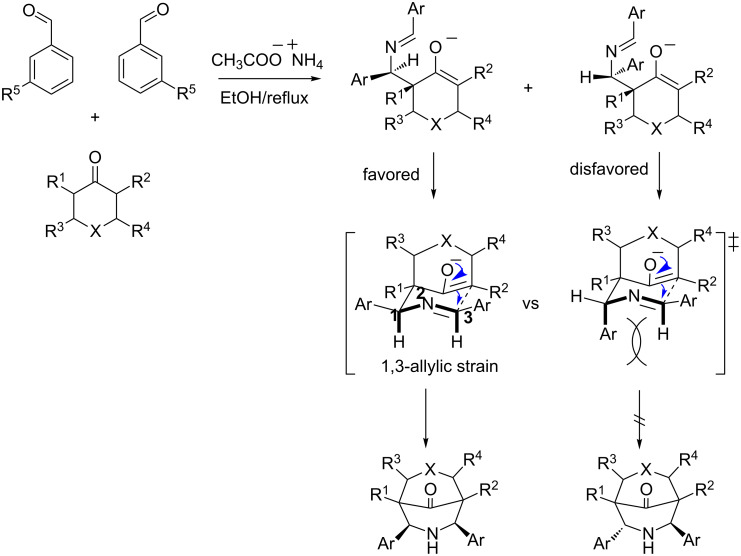
Proposed reaction mechanism for the synthesis of piperidones by the Mannich reaction. The substituents are shown in Figure 4.

**Scheme 2:**
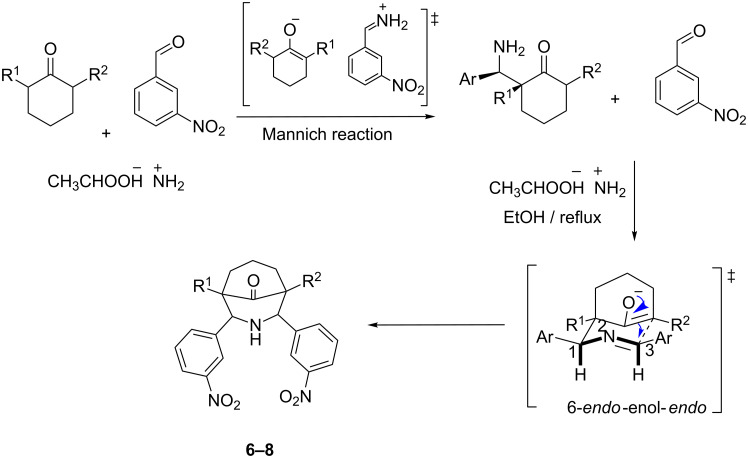
For **6**, R^1^ = R^2^ = H, for **7**, R^1^ = H, R^2^ = CH_3_, for **8**, R^1^ = R^2^ = CH_3_.

The 2,4-diphenyl-3-azabicyclo[3.3.1]nonan-9-ones were synthesized by the Mannich reaction [46–49]. As shown in Scheme 1; **1** and **2** were synthesized using ammonium acetate, benzaldehyde, and acetone or 2-butanone (4:4:1), respectively [50–52].

When we probed this reaction with 3-pentanone, the product was 3,5-dimethyl-2,6-diphenylpiperidin-4-one, while the reaction with 2-butanone gave **2** and 3-methyl-2,6-diphenylpiperidin-4-one in a ratio of 9:1 (Scheme S2, Supporting Information File 1).

Piperidones have previously been prepared using ammonium acetate, benzaldehyde, and cyclohexanone for **3**, ammonium acetate, benzaldehyde, and 2-methylcyclohexanone for **4**, and ammonium acetate, benzaldehyde, and 2,6-dimethylcyclohexanone for **5** in a 2:2:1 ratio Scheme 1 [53–56].

According to Scheme 1, two possible diastereomers could be obtained for **3**–**8**: both phenyl groups are in a *cis* orientation and both phenyl groups in a *trans* orientation. However, only the diastereomer with the phenyl groups in the *cis* orientation was obtained. This is because in the transition state (TS) for the *cis* compounds the energy of the 1H,3H-allylic strain (A^1,3^) is lower in comparison with the TS trans,1Ar,3H-allylic strain [57], which is caused by the hindrance effect of the phenyl group on C(1) with the proton on C(3).

The new compounds **6**–**8** (Scheme 2) were synthesized by the reaction between ammonium acetate, *m*-nitrobenzaldehyde, and cyclohexanone for **6**, ammonium acetate, *m*-nitrobenzaldehyde, and 2-methylcyclohexanone for **7**, and ammonium acetate, *m*-nitrobenzaldehyde, and 2,6-dimethycyclohexanone for **8**.

The proposed reaction mechanism for piperidone formation is through an aldimine, which is formed by the reaction between an aldehyde and ammonium acetate. The aldimine is then attacked by a keto–enol to form a β-aminocarbonyl, which reacts with another molecule of aldehyde to form a second aldimine. Finally, 6-*endo-*enol*-endo* intramolecular cyclization leads to piperidone formation (Scheme 2) [58–59].

### Structural and conformational analysis of piperidones **1**–**8** by NMR and X-ray diffraction

Solution characterization of **1**–**8** was performed by ^1^H and ^13^C NMR, and mass spectrometry. The H,H and H,C connectivities were determined by COSY and HSQC experiments, while the conformation was determined by nOe through t-ROESY experiments.

**Figure 5:**
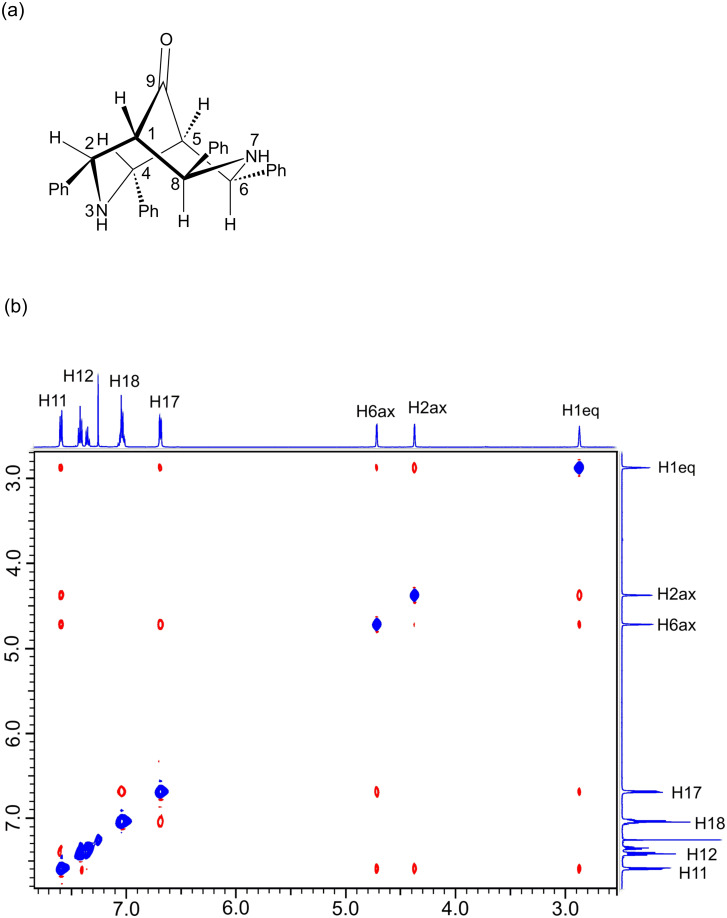
(a) Favored conformation for compound **1**, determined by nOe effect, (b) t-ROESY spectrum of **1** recorded at 500 MHz in CDCl_3_.

Figure 5 shows the ^1^H NMR spectrum of **1**. Three signals are present in the aliphatic region: H(2,4)_ax_ protons as a double signal at δ = 4.37, H(6,8)_ax_ protons as a doublet at δ = 4.72, and H(1,5)_eq_ protons shifted to δ = 2.87. The H,H coupling constants are 2.1 Hz for ^3^*J*_H(1)eq,H(2)ax_ and 3.0 Hz for ^3^*J*_H(1)eq,H(8)ax_. In the ^13^C NMR spectrum, three aliphatic signals are present at 61.80, 63.41, and 58.77 ppm, which correspond to C8, C1, and C2, respectively. A signal corresponding to the carbonyl carbon is located at 211.66 ppm.

Figure 5a shows the conformations of **1**, determined by the t-ROESY experiment. In solution, one of the six-membered rings is in the chair conformation, while the second is in the boat conformation to prevent repulsion between the LPEs of the nitrogen.

**Figure 6:**
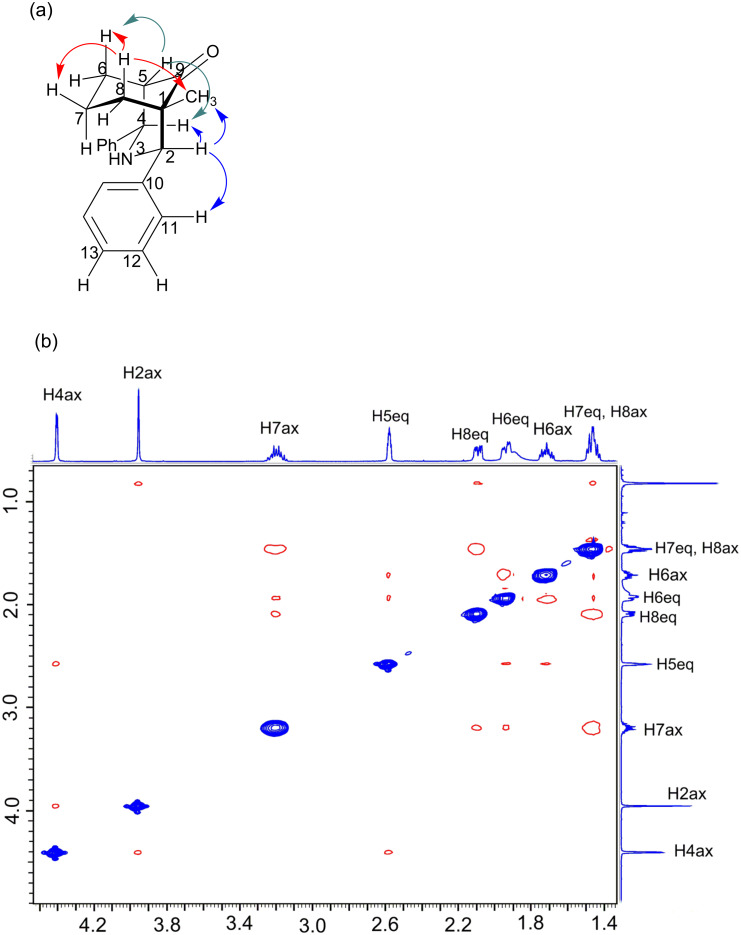
(a) Preferred conformation of **4** determined by nOe, (b) t-ROESY spectrum of **4** recorded at 500 MHz in CDCl_3_.

Although **3**, **5**, **6**, and **8** have four stereogenic centers, however, there is a mirror plane that passes through N(3), C(9), and C(7), so these compounds do not exhibit optical activity. Compounds **4** and **7** have a methyl group on the C(1) carbon, where there is no mirror plane, thereby making **4** and **7** asymmetric and they were obtained as racemic mixtures. In the ^1^H NMR spectrum of **4** (Figure 6), H(4)_ax_ is located at δ = 4.40, with a ^3^*J*_H,H_ coupling of 3.0 Hz with H(5)_eq_, which is shifted to δ = 2.57. H(2)_ax_ is present as a singlet at δ = 3.95, H(6)_ax_, H(7)_ax_, and H(8)_ax_ are located at δ = 1.71, 3.19, and 1.46 ppm, respectively, while H(6)_eq_ and H(7)_eq_ are located at δ = 1.94 and 1.45, respectively, and H(8)_eq_ shifted to δ = 2.08. H(6)_ax_ and H(8)_ax_ shifted to lower frequencies than H(6)_eq_ and H(8)_eq_ with Δδ = H(6)_eq_ − H(6)_ax_ = 0.23, while Δδ = H(8)_eq_ − H(8)_ax_ = 0.62. H(7)_ax_ is shifted to higher frequency than H(7)_eq_ with Δδ = H(7)_eq_ − H(7)_ax_ = −1.74. Table S1 (Supporting Information File 1) lists the proton chemical shifts and *^n^J*_H,H_ coupling constants.

The H,C-HSQC spectrum of **4** shows that C(1), C(2), and C(4) shifted to δ = 50.94, 71.33, and 65.06, respectively, while C(5), C(6), C(7), and C(8) shifted to δ = 54.49, 29.23, 21.54, and 36.96, respectively. The carbonyl carbon is located at 218.01 ppm. Table S2 (Supporting Information File 1) lists all of the ^13^C chemical shifts and ^1^*J*_C,H_ values. The conformation of the piperidones was determined in solution through nOe (Figure 6a). In **4**, nOe was observed among H(4)_ax_, H(5)_eq_, and H(2)_ax_, and for H(7)_ax_ with H(6)_eq_ and H(8)_eq_. Furthermore, nOe was observed among H(5)_eq_, H(6)_ax_, and H(6)_eq_ (Figure 6b). Based on the nOe values and ^3^*J*_H,H_ coupling constants, the solution conformation was determined using the Karplus curve [60]. For **4**–**8**, the six-membered rings exhibit the chair–chair conformation, with both of the phenyl rings in equatorial positions. For **1** and **2,** the six-membered rings exhibit the chair–boat conformation, because one nitrogen atom is substituted by a methyl group. As a result, LPE repulsion is absent in compounds **3**–**8**.

The solid-state conformations of the piperidones were determined by X-ray diffraction (XRD). Crystals of **1**, **3**, **5**, **6**, and **7** suitable for XRD were obtained. The crystal structures of **3** and **5** have been previously reported [47,61–63]. The crystal structures of **1**, **6**, and **7** are reported for the first time. Crystals of **1** were obtained by slow evaporation of a saturated toluene solution, and **1** crystallized in the *P*2_1_/*n* space group. The six-membered ring in the crystal structure exhibits a chair–boat conformation.

**Figure 7:**
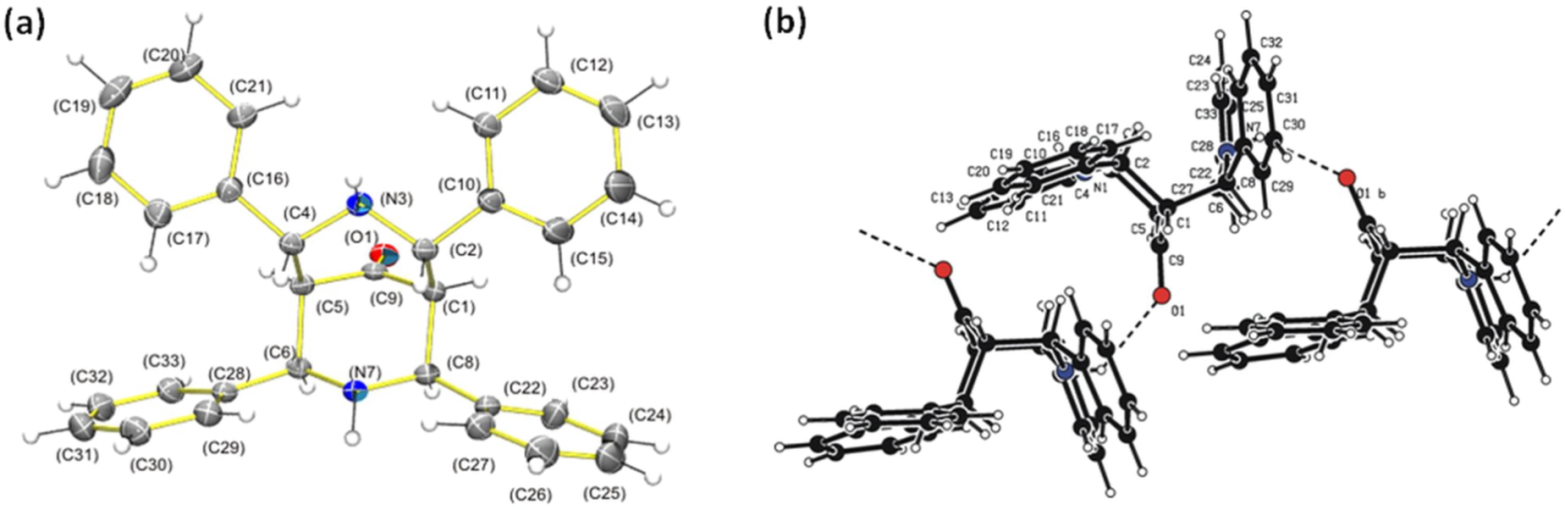
(a) ORTEP diagram of **1**. The thermal ellipsoids are drawn at the 30% probability level for all atoms other than H. (b) Crystal packing and N(3)–H(3)∙∙∙O(1) intermolecular hydrogen bonds of **1**.

For **1**, the crystal structure shows that the aromatic rings on the six-membered ring with the boat conformation are antiperiplanar to the aromatic ring on the six-membered ring with the chair conformation (Figure 7a). This geometry allowed a C–H∙∙∙π intramolecular interaction between the aromatic rings. There are also C–H∙∙∙π intermolecular interactions [61–63]. Two intermolecular hydrogen bonds are observed for N(3)–H(3)∙∙∙O(1) (Figure 7b). One hydrogen bond has a N(3) nitrogen donor (D) on molecule 1 and a carbonyl acceptor (A) on molecule 2. In the second hydrogen bond, the carbonyl on molecule 1 (A) interacts with a nitrogen N(3) (D) of molecule 3, forming chains of molecules. The D∙∙∙A distance is 2.971(4), and the N(3)–H(3)∙∙∙O(1) angle is 131°.

**Figure 8:**
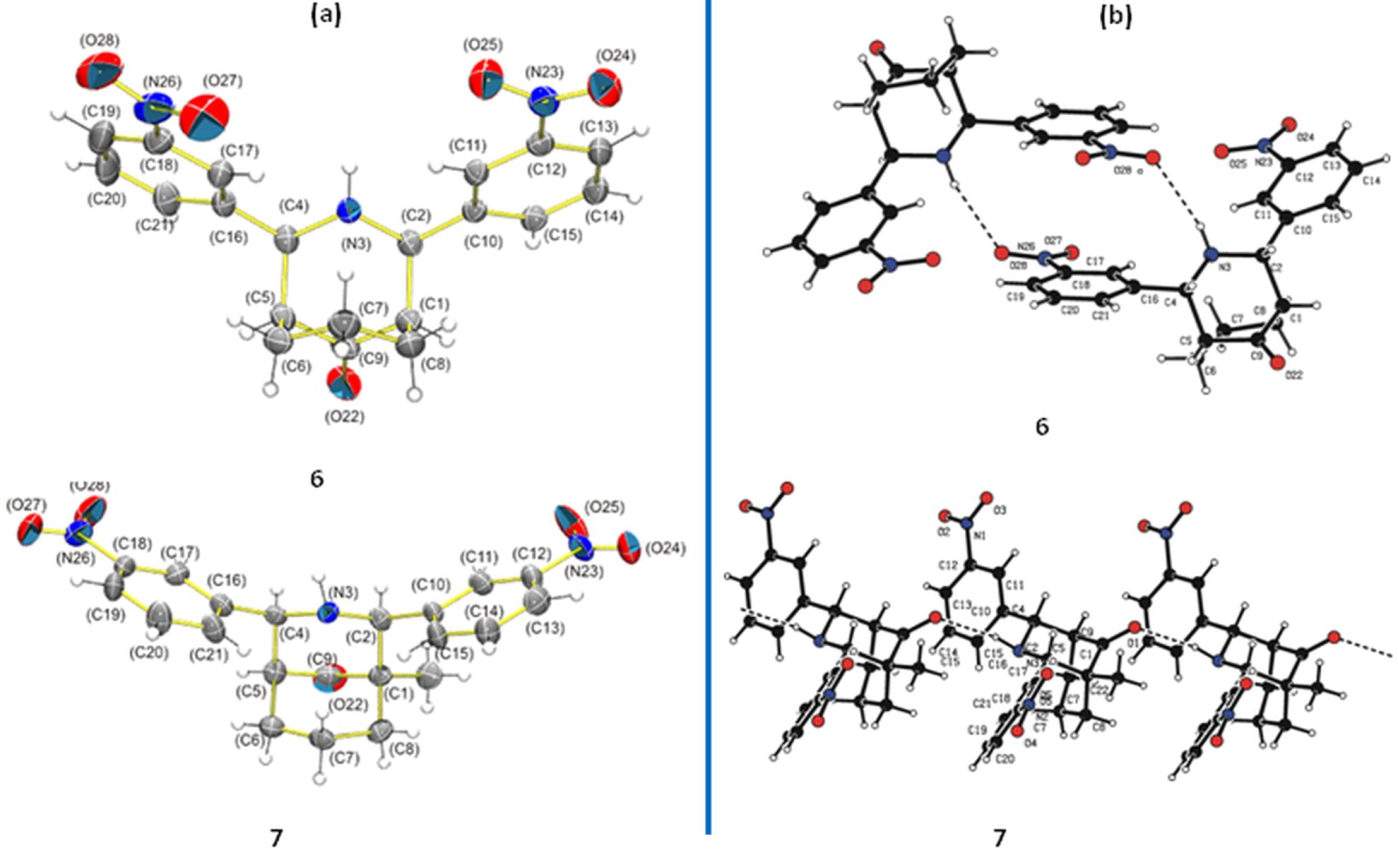
(a) ORTEP diagrams of **6** and **7**. The thermal ellipsoids are drawn at the 30% probability level for all atoms other than H. (b) N(3)–H(3)∙∙∙O(28) intermolecular hydrogen bonds of **6** and N(3)–H(3)∙∙∙O(22) intermolecular hydrogen bonds of **7**.

Crystals of **6** were obtained by slow evaporation of a saturated acetone solution. The molecule crystallized in a triclinic crystal lattice with the *P*−1 space group. Crystals of **7** were obtained by slow evaporation of a saturated toluene solution. Compound **7** crystallized in an orthorhombic crystal lattice with the non-centrosymmetric space group *Pna*2_1_. Hence, the molecule exhibits optical activity (Figure 8a).

Figure 8b shows the crystal structure of **6**, where cycles formed through the intermolecular hydrogen bond N(3)–H(3)∙∙∙O(28). The D∙∙∙A distance is 3.186(3) Å and the N(3)–H(3)∙∙∙O(28) angle is 160 ± 2°. Compound **7** formed chains of molecules by N(3)–H(3)∙∙∙O(22) hydrogen bonds with a D∙∙∙A distance of 3.261(3) Å and a N(3)–H(3)∙∙∙O(28) angle of 162 ± 1°.

### Stereoelectronic effect analysis

σ→σ*, σ→π*, nx→σ*, nx→π*, hyperconjugation, and homohyperconjugative interactions significantly affect the Fermi contact contribution to the scalar spin–spin coupling constant. Therefore, the C–H coupling constants were used to investigate the stereoelectronic effects [64–65]. The main interactions found by NBO are listed in Supporting Information File 1.

**Figure 9:**
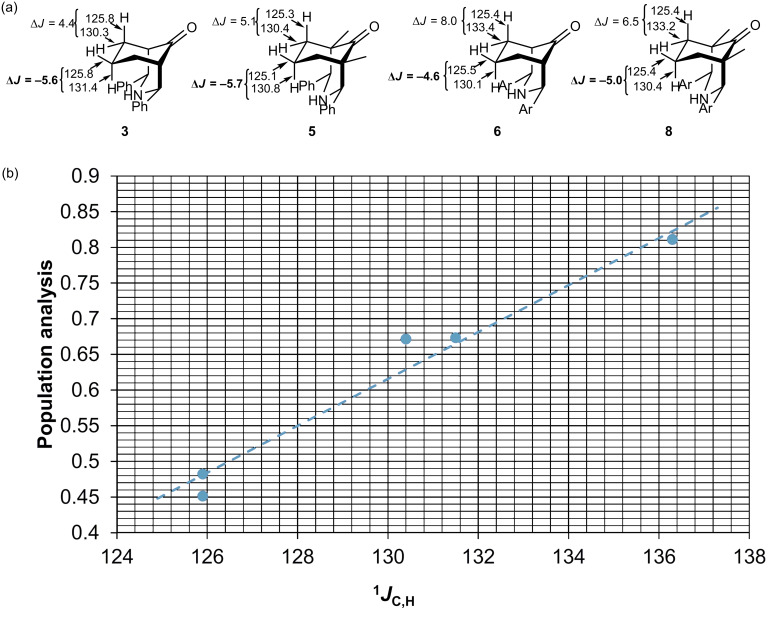
(a) ^1^*J*_C,H_ coupling constant of **3**, **5**, **6**, and **8**. (b) Plot of the population analysis versus ^1^*J*_C,H_ (slope = 0.0329 and R^2^ = 0.9748).

Hyperconjugation and homohyperconjugation in piperidones were investigated by analyzing the ^1^*J*_C,H_ coupling constants, which were measured in the ^13^C satellites in the ^1^H NMR spectrum obtained with dqf-heteronuclear pulse sequence [66–67]. Figure 9a shows the ^1^*J*_C,H_ coupling constants for **3**, **5**, **6**, and **8,** as well as the coupling constant difference between axial and equatorial protons. The Δ^1^*J*_C,H_ values (^1^*J*_C,Heq_–^1^*J*_C,Hax_) for the proton on the β-carbon with respect to the carbonyl group for **3**, **5**, **6,** and **8** are 4.4, 5.1, 8.0, and 6.5 Hz, respectively. The Δ^1^*J*_C,H_ coupling constant difference for cyclohexane is 3.9 Hz. There is a linear relationship between the ^1^*J*_C,H_ value and the population analysis using the SCF density (Figure 9b), which was estimated for compound **3**.

**Scheme 3:**
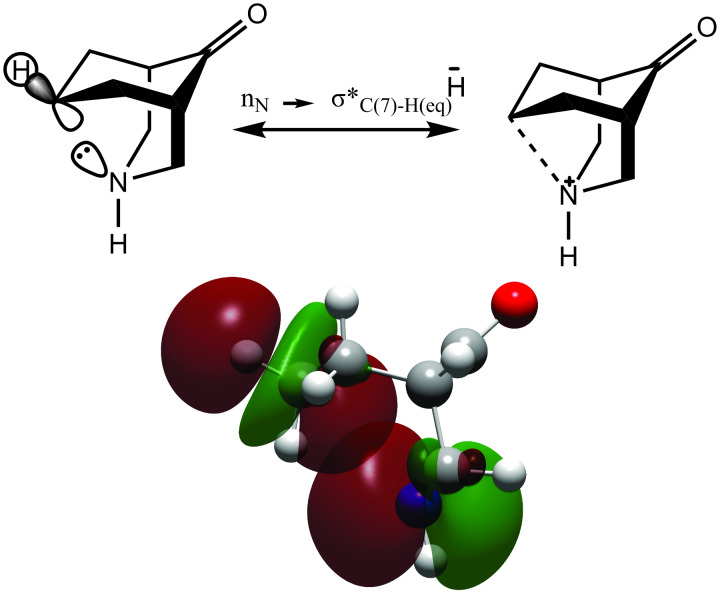
Representation of the n_N_→σ*_C–H(7)eq_ interaction. The interaction energy is 0.55 kcal/mol at the ωB97XD/6-311++G(d,p) level.

The Δ^1^*J*_C,H_ (= ^1^*J*_C,H(7)eq_ − ^1^*J*_C,H(7)ax_) value for compound **8** is −5.0 Hz, and the Δ^1^*J*_C,H_ values for **3–7** are −5.6, −5.8, −5.7, −4.6, and −3.6 Hz, respectively. The negative sign of the Δ^1^*J*_C,H_ coupling constant difference suggests that there is an effect that changes the ^1^*J*_C,H_ values. This effect is related to the proximity and geometrical relation between the LPE of nitrogen and the antibonding σ orbital C–H(7)_eq_. The hyperconjugative interaction n_N_→σ*_C–H(7)eq_ is the effect that alters the ^1^*J*_C,H_ values, causing a change in the Fermi contact term (Scheme 3). This interaction was determined in compound **3** by second-order perturbation theory analysis of the Fock matrix in the NBO basis (0.55 kcal/mol).

**Figure 10:**
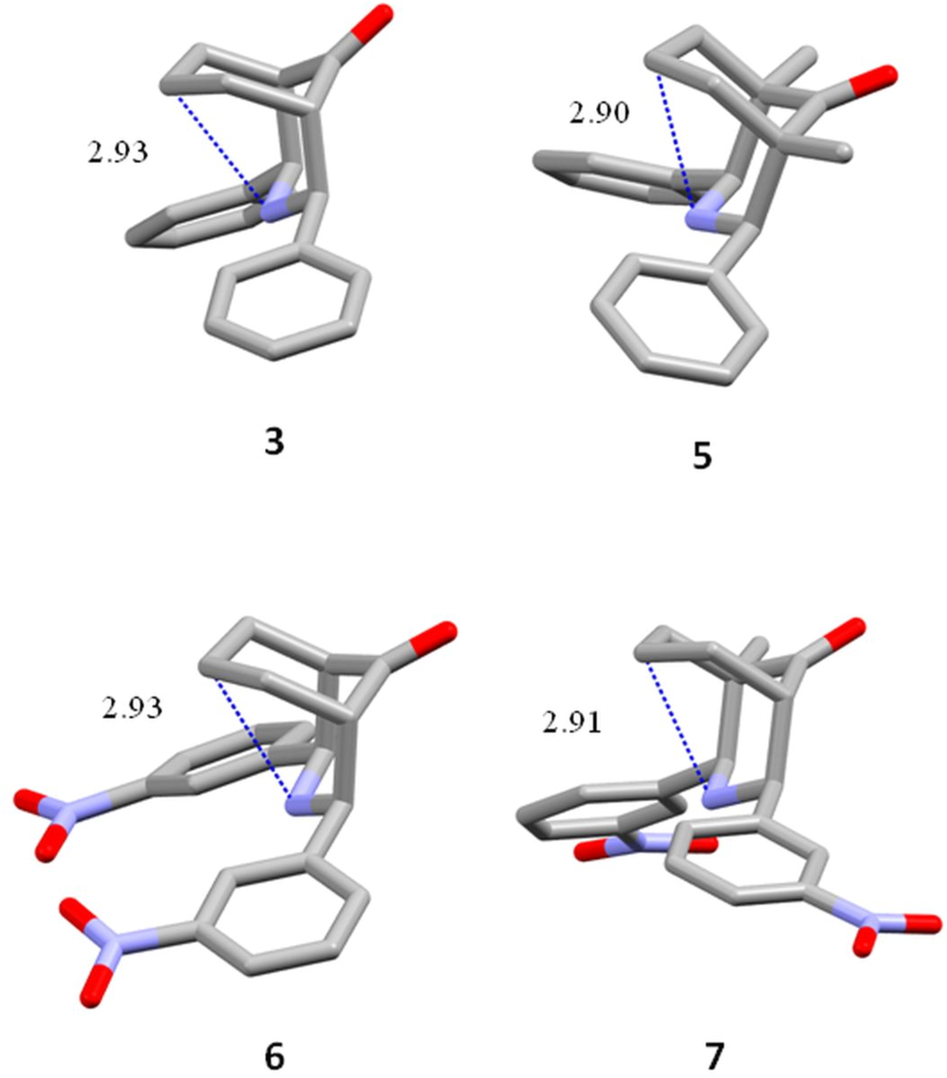
Distances between N(3) and C(7) for **3**, **5**, **6**, and **7** measured in the structures obtained by XRD.

In the crystal structures of **3**, **5**, **6**, and **7**, the geometric relationship between the LPE of nitrogen and the antibonding σ orbital C–H(7)_eq_ promotes the n_N_→σ_C–H(7)eq_ interaction, which is caused by the N(3)–C(7)–H_eq_ angle being near 160° (Table 1). Furthermore, the N(3) ∙∙∙C(7) distance was measured, and it is close to 2.9 Å (Figure 10). The distance determined from a ωB97XD/6-311++G(d,p) calculation of **3** is 2.96 Å. This value is in agreement with n_N_→σ_C–H(7)eq_ hyperconjugation. Hence, the chair–chair conformation in **3**–**8** is preferred over the chair–boat conformation in **1** and **2**.

**Table 1:** Distances (Å) of N∙∙∙C(7) and angles (°) of N(3)–C(7)–H(7)_eq_ for **3**, **5**, **6**, and **7**.

Comp.	D (Å)	angles of N(3) ∙∙∙C(7) ∙∙∙H(7)_eq_

**3**	2.935	159.24
**5**	2.907	161.65
**6**	2.935	159.85
**7**	2.913	159.85

## Conclusion

The six-membered rings in **1** and **2** prefer the chair–boat conformation because that conformation minimizes repulsion between the LPEs of both nitrogen atoms. Moreover, the six-membered rings of **3**–**8** exhibit the chair–chair conformation because of the substitution of the nitrogen atom by the methylene group (CH_2_).

The ^1^*J*_C,H_ coupling constants measured by ^1^H NMR show a linear relationship with the electron density by NBO analysis. The chemical shift difference between H(7)_ax_ and H(7)_eq_ is 1.7 ± 0.3 ppm, with H(7)_ax_ shifted to a higher frequency. The ^1^*J*_C,Heq_ coupling constant is 4.7 ± 1.1 Hz lower than the ^1^*J*_C,Hax_ coupling constant because of homohyperconjugation between the LPE of nitrogen and the C–H(7)_eq_ antibonding σ orbital (n_N_→σ*_C(7)–Heq_). This interaction indicates that the H(7)_ax_ and H(7)_eq_ chemical shift difference is because of the n_N_→σ*_C(7)–Heq_ interaction: the electronic density in the C–H(7)_eq_ bond increases and shields the proton. Moreover, this information is supported by the X-ray diffraction structures, in which the measured C(7)–N(3) distance and geometric disposition of the N(3)–C(7)–H_eq_ angle suggest that the homohyperconjugative interaction is important for the preferred conformations of 2,4-diphenyl-3-azabicyclo[3.3.1]nonan-9-ones.

## Experimental

### Spectra

The NMR spectra of **1**–**8** were recorded at 21 ± 1 °C using a Jeol ECA 500 spectrometer equipped with a 5 mm multinuclear pulse-field gradient probe. All of the spectra were recorded in CDCl_3_ solution (0.9 mmol of the compound per 0.5 mL solvent). The chemical shifts were referenced to tetramethylsilane (CH_3_)_4_Si, which served as an internal standard (δ^1^H = 0, δ^13^C = 0).

^1^H NMR spectra were recorded at 500.159 MHz using a spectral width of 9384.3 Hz, acquisition time of 6.98 s, 65536 points, 512 scans, and a recycle delay of 1 s. Fourier transformation was applied using an exponential function (line width = 0.2). ^13^C satellites were directly determined from the ^1^H NMR spectra or using the same spectral condition as the heteronuclear-double-quantum filter experiment [66–67].

^13^C NMR spectra were recorded using a single-pulse decoupling experiment both with and without nOe at 125.76 MHz using a spectral width of 31446.54 Hz, an acquisition time of 0.8 s, 32678 points, 2052 scans, and a recycle delay of 0.1 s. The non-decoupled ^13^C NMR spectra with nOe were recorded with a spectral width of 39308.17 Hz, a resolution of 0.9 Hz, and 2048 scans, and the process was performed using the sine-bell function and zero filling.

^1^H,^1^H-COSY spectra were obtained using a dqf-COSY pulse sequence with a data point matrix of 1024 × 256, a spectral width of 9384 × 9384 Hz, and a recycle delay of 1.5 s. t-ROESY spectra were obtained using a data point matrix of 1024 × 256 with a spectral width of 6354 × 6354 Hz, mixture time of 1 s, and a recycle delay of 1.5 s [66–67].

Mass spectra were recorded on an Agilent G1969 LC/MSD TOF spectrometer coupled to HPLC with electrospray ionization.

### X-ray crystal structure analysis

The crystals were mounted on a glass fiber and collected on an Enraf-Nonius CAD-4 diffractometer, a Kappa CCD with an area detector using Mo Kα (λ = 0.71073 Å) radiation at 293 K. Intensity data were collected and processed using CAD4 EXPRESS Software. The structures were solved using WinGX [68] by direct methods with SHELXS-97 [69] and refined by the full-matrix least-squares method on F2 with SHELXL-97. Note that in this report we use our crystallography results of compounds **1**, **3**, **5**, **6**, and **7**.

### Details of the calculations and computational methods

The computational chemistry calculations were performed using the Gaussian 09 package [70], and molecular visualization was performed with ChemCraft 1.7 (2013) software [71]. Geometry optimization and natural bond orbital (NBO) analysis was performed for compounds **1** and **3** using density functional theory (DFT) with the long-range corrected ωB97XD functional [72–73] and the 6-311++G(d,p) basis set. This basis set includes diffusion and polarization functions, and performs better for the description of molecular orbitals from geometry optimization and NBO analysis.

### General procedure for the synthesis of 2,4-diphenyl-3-azabicyclo[3.3.1]nonan-9-ones

The compounds **1–2** were prepared following a previously reported method, and their physical and spectroscopic properties are in good agreement with the reported values [50–52].

The synthesis of 2,4-diphenyl-3-azabicyclo[3.3.1]nonan-9-one was performed by the following method. First, 5.10 mmol of ketone, 10.2 mmol of ammonium acetate, and 10.2 mmol of benzaldehyde or *m*-nitrobenzaldehyde were added to a 100 mL flask, followed by addition of 30 mL of ethanol. Second, the mixture was stirred and heated to reflux, it was monitored by thin-layer chromatography, and was stopped when the reaction was completed. Next, the mixture was cooled, 30 mL of water was added, and the mixture was neutralized using aqueous NaOH, followed by extraction using dichloromethane (3 × 25 mL). Finally, the organic phases were combined and dried over MgSO_4_, and the solvent was removed. The compounds were purified by recrystallization in 2:1 acetone–methanol. Yields were 60–80%.

The physical and spectroscopic properties of compounds **3**–**5** are in agreement with previous reports [53–56].

**2,4-Bis(3-nitrophenyl)-3-azabicyclo[3.3.1]nonan-9-one (6).** White solid, Yield: 73%, by using the general procedure 5.10 mmol of cyclohexanone and 10.2 mmol of ammonium acetate, and 10.2 mmol of *m*-nitrobenzaldehyde were added to the reaction. ESIMS–TOF: *m*/*z* for C_20_H_19_N_3_O_5_ [M + H]^+^ calcd: 382.1397, found: 382.1395; ^1^H NMR (500 MHz, CDCl_3_) δ 8.41 (s, 2H, H11, H17), 8.20 (d, *J* = 8.2 Hz, 2H, H13, H19), 7.92 (d, *J* = 7.6 Hz, 2H, H15, H21), 7.64 ( t, *J* = 7.6 Hz, 2H, H14, H20), 4.58 (d *J* = 1.9 Hz, 2H, H2, H4), 2.82 ( dtt, *J* = 13.3, 13.2, 6.2 Hz, 1H, H7*ax*), 2.56 (d, *J* = 1.9 Hz, 2H, H1, H5), 1.86 (dd, *J* = 13.6, 6.2 Hz, 2H, H6*ec*, H8*ec*), 1.78 (tt, *J* = 13.6, 6.2 Hz, 2H, H6*ax*, H8*ax*), 1.49 (dd, *J* = 13.2, 6.2 Hz, 1H, H7*ec*); ^13^C NMR (500 MHz, CDCl_3_) δ 21.18 (C7), 28.92 (C6, C8), 53.24 (C1, C5), 64.02 (C2-NH, C4-NH), 121.90 (C13, C19), 123.08 (C11, C17) 129.94 (C14, C20), 133.05 (C15, C21), 142.82 (C10, C16), 148.70 (C12-C18), 214.92 (C=O).

**1-Methyl-2,4-bis(3-nitrophenyl)-3-azabicyclo[3.3.1]nonan-9-one (7).** Yellow solid, Yield: 72%, by using the general procedure 5.10 mmol of 2-methylcyclohexanone and 10.2 mmol of ammonium acetate, and 10.2 mmol of *m*-nitrobenzaldehyde were added to the reaction. ESIMS–TOF: *m*/*z* for C_21_H_21_N_3_O_5_ [M + H]^+^ calcd: 396.1553, found: 396.1558; ^1^H NMR (500 MHz, CDCl_3_) δ 8.39 (s, 1H, H11), 8.34 (s, 1H, H17), 8.19 (dd, *J* = 8.1, 1.9 Hz, 2H, H13), 8.15 (dd, *J* = 8.1, 1.9 Hz, 2H, H19), 7.94 (d, *J* = 7.5 Hz, 1H, H15), 7.58–7.63 (m, 2H, H14, H20), 7.87 (d, *J* = 7.6 Hz, 1H, H21), 4.56 (d, *J* = 2.3 Hz, 1H, H4), 4.12 (s, 1H, H2), 3.11 (dtt, *J* = 13.4, 13.2, 6.3 Hz, 1H, H7*ax*), 2.63 (dd, *J* = 2.9, 2.3 Hz, 1H, H5), 2.01 ( ddd, *J* = 13.6, 5.6, 2.3 Hz, 1H, H8*ec*), 1.84 (ddd, *J* = 13.8, 6.3, 2.3 Hz, 1H, H6*ec*), 1.76 ( tt, *J* = 13.4, 6.3 Hz, H6*ax*), 1.46–1.45 (m, 2H, H7*ec*, H8*ax*), 0.82 (s, 3H, Me); ^13^C NMR (500 MHz, CDCl_3_) δ 20.25 (Me), 21.45 (C7), 29.01 (C6), 36.74 (C8), 50.55 (C1), 53.68 (C5), 70.42 (C2), 121.77 (C17), 122.92 (C19), 123.35 (C13), 123.85 (C11), 129.42 (C20), 129.86 (C14), 133.03 (C21), 135.21 (C15), 141.37 (C16) 142.93 (C10), 148.26 (C18), 148.61 (C12), 215.46 (C=O).

**1,5-Dimethyl-2,4-bis(3-nitrophenyl)-3-azabicyclo[3.3.1]nonan-9-one (8).** White solid, Yield: 64%, by using the general procedure 5.10 mmol of 2,6-dimethylcyclohexanone and 10.2 mmol of ammonium acetate, and 10.2 mmol of *m*-nitrobenzaldehyde were added to the reaction. ESIMS-TOF: *m*/*z* for C_22_H_23_N_3_O_5_ [M + H]^+^ calcd: 410.1710, found: 410.1713; ^1^H NMR (500 MHz, CDCl_3_) δ 7.53 (d, *J* = 7.4 Hz, 4H, H11, H15, H17, H21), 7.36 (t, *J* = 7.4 Hz, 4H, H12, H14, H18, H20), 7.30 (t, *J* = 7.4 Hz, 2H, H13, H19), 3.35 (s, 2H, H2, H4), 3.03 ( dtt, *J* = 13.8, 13.2, 6.8 Hz, 1H, H7*ax*), 1.61 (dd, *J* = 13.8, 6.8 Hz, 2H, H6*ec*, H8*ec*), 1.11 (H6*ax* ) 0.81 (H8*ax*), 0.69 (s 6H, Me); ^13^C NMR (500 MHz, CDCl_3_) δ 20.26 (Me), 21.10 (C7), 36.40 (C6, C8), 50.15 (C1, C5), 70.22 (C2-NH, C4-NH), 123.35 (C13, C19), 123.85 (C11, C17), 129.42 (C14, 20), 135.21 (C15, C21), 142.93 (C10, C16), 148.61 (C12, C18), 215.46 (C=O).

## Supporting Information

Crystallographic data of the structures reported in this paper have been deposited with the Cambridge Crystallographic Data Centre with supplementary publication numbers CCDC 928314 (**1**), 928315 (**6**), and 933224 (**7**). These data can be obtained free of charge from The Cambridge Crystallographic Data Centre via http://www.ccdc.cam.ac.uk/data_request/cif.

File 1 Additional schemes, figures, theoretical, spectra, and crystallographic data.
