# Supplementary material for: Investigation of the role of stereoelectronic effects in the conformation of piperidones by NMR spectroscopy and X-ray diffraction
Source: Beilstein J Org Chem. 2015 Oct 22;11:1973–84. doi: 10.3762/bjoc.11.213 (PMC4660988; doi:10.3762/bjoc.11.213)
Supplement: File 1 — Additional schemes, figures, theoretical, spectra, and crystallographic data. [file Beilstein_J_Org_Chem-11-1973-s001.pdf]

Supporting Information

for

Investigation of the role of stereoelectronic effects in the conformation of piperidones by NMR spectroscopy and X-ray diffraction

Cesar Garcias-Morales, David Ortegón-Reyna and Armando Ariza-Castolo*

Address: Departamento de Química, Centro de Investigación y de Estudios Avanzados del Instituto Politécnico Nacional, Avenida Instituto Politécnico Nacional 2508 Colonia San Pedro Zacatenco, C.P. 07360, México, D.F., MEXICO

Email: Armando Ariza-Castolo - aariza@cinvestav.mx
*Corresponding author

Additional schemes, figures, theoretical, spectra, and crystallographic data

Content:

Theoretical calculations summary
Compound **3**
Cartesian coordinates s3
Natural population analysis s4
Perturbation theory energy analysis of Fock matrix s5
Compound **1**
Cartesian Coordinates s6
Natural population analysis s8
Perturbation theory energy analysis of Fock matrix s9
Crystallography data
Compound **1** s10
Compound **6** s10
Compound **7** s10
NMR Data:
Table S1. ^{1}H NMR data: chemical shift (δ) and $^{n}J_{H,H}$ (Hz) for compound **1-8** in CDCl3 s11
Table S2. ^{13}C NMR data: chemical shift (δ) and $^{1}J_{C,H}$ (Hz) for compound **1-8** in CDCl3 s12
Schemes:
Scheme S1. (a) Stabilization of positive charges by the homohyperconjugation effect in organometallic molecules. (b) Stabilization of conformation by LPE (N, O and S) homohyperconjugation through W arrangement and Plough effect. s13
Scheme S2. Reaction mechanism proposed for the synthesis of piperidones **1** and **2** by double Mannich reaction s14

Scheme S3. Reaction mechanism proposed for the synthesis of piperidones **3** to **8** by Mannich reaction s15
Scheme S4. A(1,3) allylic strain. s16
Scheme S5. (a) preferred conformation for the compound **1** and **2**. (b) dqf- COSY spectrum of compound **1** in $CDCl_3$. (c) t-ROESY spectrum of compound **1** in $CDCl_3$. s16
Scheme S6. NMR characterization of compound **4**. (a) ^1H spectrum in $CDCl_3$. (b) dqf-COSY spectrum. (c) HSQC spectrum$_3$. s17
Scheme S7. Determination of the conformation of piperidones by t-ROESY spectrum s18
Scheme S8. (a) ORTEP diagram of compounds **1**, thermal ellipsoids are drawn at 30% probability level for all atoms other than H. (b) crystal packing and C(15)···H(15)···π inter and intramolecular interaction of compound **1**. s19
Scheme S9. (a) ORTEP diagram of compounds **6** and **7**, thermal ellipsoids are drawn at 30% probability level for all atoms other than H. (b) Intermolecular hydrogen bonds of compound **6** and **7** s20
Scheme S10. $^1J_{C,H}$ coupling constant of compound **3**, **5**, **6** and **8** s21
Scheme S11. Schematic representation of $n_X \rightarrow \sigma^*_{C7\text{-}H_{eq}}$ Hyperconjugation in the piperidones **3-8** s21
Scheme S12. Distance between N(3) and σ*C-H orbital of compound **3**, **5**, **6** and **7,** measured by X-ray s22
Select spectra
^1H NMR spectrum of **1** s23
^{13}C NMR spectrum of **1** s24
^1H NMR spectrum of **2** s25
^{13}C NMR spectrum of **2** s26
^1H spectrum of compound **3** s27
^{13}C spectrum of compound **3** s28
^1H,^1H COSY spectrum of compound **3** s29
dqf spectrum of compound **3** s30
^1H NMR spectrum of compound **4** s31
^{13}C NMR spectrum of compound **4** s32
HSQC spectrum of compound **4** s33
t-ROESY spectrum of compound **4** s34
^1H spectrum of compound **5** s35
^{13}C spectrum of compound **5** s36
dqf spectrum of compound **5** s37
^1H spectrum of compound **6** s38
^{13}C spectrum of compound **6** s39
^1H NMR spectrum of compound **7** s40
^{13}C NMR spectrum of compound **7** s41
^1H-^1H COSY spectrum of compound **7** s42
HSQC spectrum of compound **7** s43
DQF spectrum of compound **7** s44

THEORETICAL CALCULATIONS SUMMARY

Compound 3:

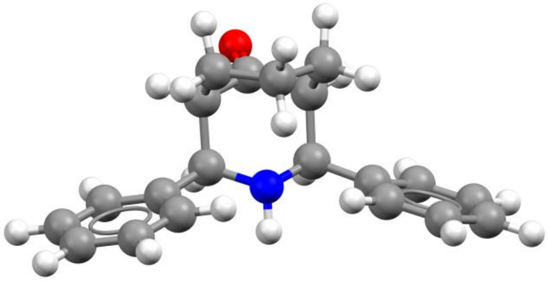

Cartesian Coordinates

Atom	X	Y	Z
C1	1.248451	1.688571	0.067809
C2	1.227609	0.388289	-0.786319
C4	-1.22763	0.388297	-0.786296
C5	-1.248451	1.688559	0.067858
C6	-1.275225	1.482261	1.596693
C7	0.00005	0.844856	2.161153
C8	1.275296	1.482286	1.596647
C9	-0.000007	2.46291	-0.288211
H1eq	2.118875	2.273235	-0.238543
H2ax	1.289286	0.726162	-1.836822
H3	-0.000019	-1.203281	-1.109553
H4ax	-1.289326	0.726191	-1.836795
H5eq	-2.118891	2.27322	-0.238454
H6ax	-1.417508	2.470693	2.048846
H6eq	-2.151439	0.886662	1.868016
H7ax	0.000066	-0.221612	1.939009
H7eq	0.000069	0.947634	3.250751
H8ax	1.417576	2.470728	2.048789
H8eq	2.151529	0.886696	1.867929
N3	-0.000014	-0.359509	-0.547615
O9	-0.000025	3.551449	-0.81209
C10	2.450483	-0.461361	-0.514087
C11	3.646217	-0.163805	-1.167052
C12	4.801068	-0.886486	-0.899218
C13	4.773668	-1.925767	0.024532
C14	3.585269	-2.234243	0.674369
C15	2.431032	-1.505645	0.40814
C16	-2.450506	-0.461358	-0.514077
C17	-3.64628	-0.163699	-1.166921

C18	-4.801122	-0.886395	-0.899087
C19	-4.773672	-1.925797	0.024527
C20	-3.585229	-2.234378	0.674231
C21	-2.431	-1.505763	0.408008
H11	3.670975	0.642357	-1.894557
H12	5.722583	-0.642863	-1.416199
H13	5.673966	-2.492683	0.233242
H14	3.555276	-3.043893	1.395221
H15	1.504463	-1.742536	0.917638
H17	-3.671079	0.642557	-1.894315
H18	-5.722675	-0.64269	-1.415966
H19	-5.673962	-2.492724	0.233229
H20	-3.555192	-3.044123	1.394971
H21	-1.504392	-1.742751	0.917388

Natural population analysis in ωB97XD/6-311++G(d,p) level theory.

Atom	Mulliken atomic charges	Natural charge	core	Valence	Rydberg	Total
**C1**	0.218704	-0.30643	1.99906	4.28539	0.02197	6.30643
**C2**	-0.041174	-0.01803	1.99911	3.99161	0.02730	6.01803
**C4**	-0.041172	-0.01744	1.99911	3.99159	0.02674	6.01744
**C5**	0.218709	-0.30643	1.99906	4.28540	0.02197	6.30643
**C6**	-0.681972	-0.38763	1.99926	4.37394	0.01443	6.38763
**C7**	-0.128455	-0.39507	1.99927	4.38278	0.01302	6.39507
**C8**	-0.681931	-0.38763	1.99926	4.37393	0.01443	6.38763
**H1eq**	0.290847	0.23224	0.00000	0.76542	0.00234	0.76776
**H2ax**	0.255559	0.18168	0.00000	0.81565	0.00266	0.81832
**H4ax**	0.255564	0.18168	0.00000	0.81565	0.00267	0.81832
**H5eq**	0.290840	0.23224	0.00000	0.76542	0.00234	0.76776
**H6ax**	0.213837	0.20509	0.00000	0.79309	0.00182	0.79491
**H6eq**	0.171444	0.22001	0.00000	0.77819	0.00180	0.77999
**H7ax**	0.080061	0.20947	0.00000	0.78838	0.00215	0.79053
**H7eq**	0.220823	0.20328	0.00000	0.79479	0.00193	0.79672
**H8ax**	0.213841	0.20508	0.00000	0.79310	0.00182	0.79492
**H8eq**	0.171443	0.21999	0.00000	0.77819	0.00182	0.78001

Perturbation theory energy analysis of Fock Matrix

NBO interaction	E(2) [kcal/mol]	E(j)-E(i) [a.u.]	F(i,j) [a.u.]
$n_N \rightarrow \sigma^*_{C7Heq}$	0.55	0.89	0.02
$n_N \rightarrow \sigma^*_{C7Hax}$			
$\sigma_{C7Heq} \rightarrow \sigma^*_{C6C5}$	3.87	1.03	0.056
$\sigma_{C6C5} \rightarrow \pi^*_{CO}$	3.89	0.8	0.05
$\sigma_{C6C5} \rightarrow \pi^*_{CO}$	2.45	1.41	0.053
$\sigma_{C6Heq} \rightarrow \sigma^*_{C5C9}$	3.06	1.06	0.051
$\sigma_{C5C9} \rightarrow \sigma^*_{CO}$	0.93	1.44	0.033
$\sigma_{C6Hax} \rightarrow \sigma^*_{C5C4}$	4.71	1.01	0.062
$\sigma_{C7Hax} \rightarrow \sigma^*_{C6Hax}$	3.01	1.06	0.051
$\sigma_{C4C5} \rightarrow \pi^*_{CO}$	4.78	0.8	0.055
$\sigma_{C4C5} \rightarrow \sigma^*_{CO}$	2.14	1.4	0.049

Compound 1:

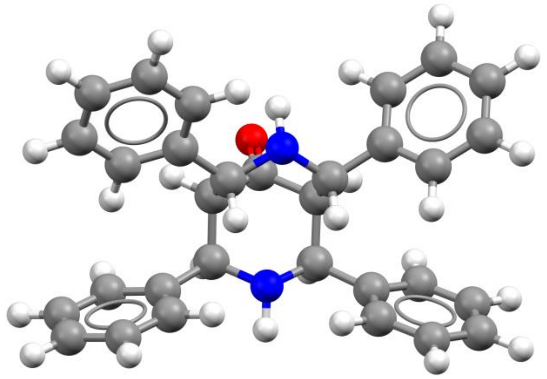

Cartesian Coordinates

Atom	X	Y	Z
C1	-1.243491	-0.113438	1.251766
C2	-1.136332	-1.636654	0.973133
C4	1.299463	-1.484838	1.053066
C5	1.240723	0.062772	1.240105
C6	1.140814	0.864805	-0.107886
C8	-1.285169	0.740091	-0.066921
C9	-0.022731	0.307571	2.030326
H1eq	-2.130018	0.067402	1.858983
H2ax	-1.187625	-2.138501	1.955315
H3	0.197201	-2.903095	0.09021
H4ax	1.30273	-1.91439	2.070501
H5eq	2.092893	0.382989	1.839914
H6ax	1.120411	0.126945	-0.915643
H7	-0.139347	2.3673	0.441115
H8ax	-1.266132	0.050218	-0.912243
N3	0.126492	-1.914697	0.304188
N7	-0.11546	1.599516	-0.223812
O9	-0.05136	0.762669	3.148714
C10	-2.323289	-2.101459	0.152445
C11	-3.57128	-2.204219	0.766862
C12	-4.697492	-2.5491	0.034268
C13	-4.59099	-2.807111	-1.328903
C14	-3.3512	-2.719839	-1.946861
C15	-2.223852	-2.366712	-1.211108
C16	2.580644	-1.920677	0.380644
C17	3.71843	-2.121612	1.162646
C18	4.937986	-2.429631	0.575837
C19	5.033826	-2.550572	-0.806658
C20	3.901522	-2.372727	-1.591077

C21	2.681025	-2.061933	-1.00169
C22	-2.565202	1.538207	-0.138267
C23	-3.534903	1.228721	-1.086931
C24	-4.732308	1.934867	-1.131341
C25	-4.971449	2.96035	-0.225753
C26	-4.01073	3.271053	0.731337
C27	-2.819587	2.559992	0.777522
C28	2.329298	1.77856	-0.368293
C29	3.631487	1.361577	-0.084463
C30	4.719886	2.173455	-0.374473
C31	4.529499	3.423422	-0.951675
C32	3.23979	3.845308	-1.244912
C33	2.150371	3.028509	-0.961482
H11	-3.663717	-2.001935	1.829885
H12	-5.660706	-2.617617	0.527029
H13	-5.470235	-3.077716	-1.902616
H14	-3.256464	-2.920815	-3.008354
H15	-1.261346	-2.279672	-1.701818
H17	3.647886	-2.019807	2.241659
H18	5.814187	-2.577186	1.197254
H19	5.985567	-2.786721	-1.268761
H20	3.968249	-2.470994	-2.668734
H21	1.800744	-1.920086	-1.617799
H23	-3.357356	0.41948	-1.788176
H24	-5.479296	1.679877	-1.874854
H25	-5.904633	3.511422	-0.259885
H26	-4.193806	4.063017	1.449038
H27	-2.091487	2.793511	1.549596
H29	3.811092	0.388219	0.355714
H30	5.721339	1.823614	-0.14876
H31	5.378938	4.059262	-1.17513
H32	3.075669	4.814407	-1.703406
H33	1.150357	3.353367	-1.219965

Natural population analysis in ωB97XD/6-311++G(d,p) level theory.

Atom	Mulliken atomic charges	Natural charge	core	Valence	Rydberg	Total
C1	-0.020307	-0.32407	1.99903	4.29887	0.02617	6.32407
C2	-0.282628	-0.01213	1.99911	3.98868	0.02435	6.01213
C4	-0.245294	-0.01732	1.9991	3.99145	0.02677	6.01732
C5	0.089815	-0.34843	1.99909	4.31377	0.03557	6.34843
C6	-0.346836	0.08808	1.99913	3.88578	0.02701	5.91192
C8	0.045344	-0.04055	1.99912	4.00347	0.03796	6.04055
C9	-0.615489	0.61974	1.99913	3.33092	0.05021	5.38026
H1eq	0.330551	0.24269	0	0.75538	0.00193	0.75731
H2ax	0.270595	0.18705	0	0.8103	0.00265	0.81295
H3	0.33721	0.37353	0	0.62336	0.00312	0.62647
H4ax	0.250479	0.18425	0	0.81301	0.00274	0.81575
H5eq	0.335355	0.24188	0	0.75534	0.00278	0.75812
H6ax	0.13706	0.21273	0	0.78296	0.00431	0.78727
H7	0.222254	0.35553	0	0.64042	0.00405	0.64447
H8ax	0.201887	0.2181	0	0.77855	0.00336	0.7819

Perturbation theory energy analysis of Fock Matrix

NBO interaction	E(2) [kcal/mol]	E(j)-E(i) [a.u.]	F(i,j) [a.u.]
$n_{N3}\rightarrow\sigma^*_{C1C2}$	1.41	0.83	0.031
$n_{N3}\rightarrow\sigma^*_{C5C4}$	2	0.82	0.037
$n_{N3}\rightarrow\sigma^*_{C4H4ax}$	10.53	0.72	0.079
$n_{N3}\rightarrow\sigma^*_{C4C16}$	0.86	0.88	0.025
$n_{N3}\rightarrow\sigma^*_{C2H2ax}$	9.89	0.87	0.084
$n_{N3}\rightarrow\sigma^*_{C2C10}$	1.07	0.89	0.028
$n_{N3}\rightarrow\sigma^*_{C25C24}$	0.81	0.3	0.014
$n_{N3}\rightarrow\sigma^*_{C27C26}$	1.42	0.2	0.015
$n_{N7}\rightarrow\sigma^*_{C1C8}$	11.54	0.82	0.088
$n_{N7}\rightarrow\sigma^*_{C5C6}$	0.91	0.48	0.019
$n_{N7}\rightarrow\sigma^*_{C6H6ax}$	1.99	0.93	0.039
$n_{N7}\rightarrow\sigma^*_{C8N7}$	2.97	0.78	0.044
$n_{N7}\rightarrow\sigma^*_{C8C22}$	0.86	0.92	0.025
$n_{N7}\rightarrow\sigma^*_{C4H4ax}$	7.57	0.73	0.067
$n_{N7}\rightarrow\sigma^*_{C4C16}$	0.51	0.89	0.019
$n_{N7}\rightarrow\sigma^*_{N3H3}$	0.6	0.89	0.021
$\sigma_{N3H3}\rightarrow\sigma^*_{C2C11}$	3.41	1.14	0.056
$\sigma_{N3H3}\rightarrow\sigma^*_{C3C9}$	3.42	1.13	0.056
$\sigma_{N3H3}\rightarrow\pi^*_{C5O8}$	1.3	0.21	0.015
$\sigma_{C4H4ax}\rightarrow\sigma^*_{C5C6}$	6.08	0.67	0.057
$\sigma_{C4H4ax}\rightarrow\pi^*_{C9O9}$	3.72	0.09	0.017
$\sigma_{C2H2ax}\rightarrow\sigma^*_{C1C8}$	3.94	1.01	0.057
$\sigma_{C2H2ax}\rightarrow\pi^*_{C9O9}$	7.54	0.09	0.024
$n_{O9}\rightarrow\sigma^*_{C1C9}$	2.54	1.26	0.051
$\pi_{O9}\rightarrow\sigma^*_{C1C9}$	24.43	0.83	0.129
$\pi_{O9}\rightarrow\sigma^*_{C5C6}$	1.38	0.43	0.022
$\sigma_{C5H5eq}\rightarrow\sigma^*_{C9O9}$	0.64	1.3	0.026
$\sigma_{C5H5eq}\rightarrow\pi^*_{C9O9}$	1.24	0.09	0.01
$\sigma_{C1H1eq}\rightarrow\sigma^*_{C9O9}$	0.68	1.3	0.027
$\sigma_{C8H8ax}\rightarrow\sigma^*_{N7H7}$	4.58	1.07	0.063
$\sigma_{C6H6ax}\rightarrow\sigma^*_{N7H7}$	4.85	1.05	0.064
$\sigma_{C6H6ax}\rightarrow\pi^*_{C9O9}$	57.12	0.06	0.052

Crystallography

The crystallographic data for compounds **1, 6** and **7** have been deposited in the Cambridge Crystallographic Data Centre with the deposition numbers CCDC 928314 (**1**), 928315 (**6**), 933224 (**7**), respectively. These data can be obtained free of charge from the Cambridge Crystallographic Data Centre via www.ccdc.cam.ac.uk/data request/cif.

Crystallographic data for 1 (2,4,6,8-tetraphenyl-3,7-diazabicyclo[3.3.1]nonan-9-one): chemical formula: $C_{31}H_{28}N_2O$; formula weight (M): 444.55; temperature: 293(2) K; monoclinic; P21/n; a = 13.986(2) Å, b = 10.039(5) Å, c = 17.529(4) Å, α = 90.00°, β = 104.99(3)°, γ = 90.00°; V = 2377.4 Å^3; Z = 4; Density (calculated) = 1.320 Mg/m^3; number of reflections collected: 16 119; Final R indices [I>2sigma(I)] R1 = 0.0539, wR2 = 0.1350; Data / restraints / parameters = 5357 / 0 / 419, Goodness-of-fit on F^2 = 1.048.

Crystallographic data for 6 (2,4-bis(3-nitrophenyl)-3-azabicyclo[3.3.1]nonan-9-one): chemical formula:$C_{20}H_{19}N_3O_5$; formula weight (M): 381.38; temperature: 293(2) K; Triclinic; P-1; a = 7.226 (4), b = 8.2780(12), c = 16.0640(10) Å, α = 96.20(2) Å, β = 91.364(1) Å, γ = 99.900(1) Å; V = 940.2(5) Å^3; Z = 2; Density (calculated) = 1.347 Mg/m^3; number of reflections collected: 6 469; Final R indices [I>2sigma(I)] R1 = 0.0589, wR2 = 0.1366; Data / restraints / parameters = 4159 / 0 / 329, Goodness-of-fit on F^2 = 1.048.

Crystallographic data for 7 (1-methyl-2,4-bis(3-nitrophenyl)-3-azabicyclo[3.3.1]nonan-9-one): chemical formula:$C_{21}H_{21}N_3O_5$; formula weight (M): 395.4; temperature: 293(2) K; Orthorhombic; Pna21; a = 6.861 (4), b = 11.744(5), c = 23.713(2) Å, α = 90.0 Å, β = 90.0 Å, γ = 90.0 Å; V = 1910.7(14) Å^3; Z = 4; Density (calculated) = 1.375 Mg/m^3; number of reflections collected: 14 713; Final R indices [I>2sigma(I)] R1 = 0.0619, wR2 = 0.1607; Data / restraints / parameters = 3314 / 1 / 278, Goodness-of-fit on F^2 = 1.073.

Table S1. ^{1}H NMR data: chemical shift (δ) and $^nJ_{H,H}$ (Hz) for compounds **1–8** in $CDCl_3$.

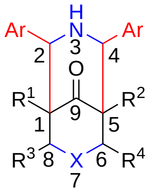

Comp.	X	Ar	R[1]	R[2]	R[3]	R[4]
1	NH	C_6H_5	H	H	C_6H_5	C_6H_5
2	NH	C_6H_5	H	CH_3	C_6H_5	C_6H_5
3	CH_2	C_6H_5	H	H	H	H
4	CH_2	C_6H_5	H	CH_3	H	H
5	CH_2	C_6H_5	CH_3	CH_3	H	H
6	CH_2	$3NO_2-C_6H_4$	H	H	H	H
7	CH_2	$3NO_2-C_6H_4$	H	CH_3	H	H
8	CH_2	$3NO_2-C_6H_4$	CH_3	CH_3	H	H

	1	2	3	4	5	6	7	8
$\delta_{H(1)eq}$	2.87	-	2.48	-	-	2.56	-	-
$\delta_{H(2)ax}$	4.37	5.3	4.41	3.95	3.93	4.58	4.12	3.96
$\delta_{H(4)ax}$	4.37	4.76	4.41	4.40	3.93	4.58	4.56	3.96
$\delta_{H(5)eq}$	2.87	3.00	2.48	2.57	-	2.56	2.63	NA
$\delta_{H(6)ax}$	4.72	4.37	1.70	1.71	1.50	1.78	1.76	1.56
$\delta_{H(6)eq}$	-	-	1.93	1.94	2.11	1.86	1.84	2.04
$\delta_{H(7)ax}$	-	-	2.9	3.19	3.49	2.82	3.11	3.49
$\delta_{H(7)eq}$	-	-	1.39	1.45	1.49	1.49	1.52	1.52
$\delta_{H(8)ax}$	4.72	3.86	1.70	1.46	1.5	1.78	1.54	1.56
$\delta_{H(8)eq}$	-	-	1.93	2.08	2.11	1.86	2.01	2.04
δ_{Me}	-	0.65	-	0.81	0.84	-	0.82	0.85
$^3J_{H(1)eq,H(2)ax}$	2.1	-	1.8	-	-	1.8	-	-
$^3J_{H(1)eq,H(8)}$	3.0	-	1.5	-	-	1.4	-	-
$^3J_{H(4)ax,H(5)eq}$	2.1	2.0	1.8	3.0	-	1.8	2.9	-
$^3J_{H(5)eq,H(6)}$	3.0	3.4	1.5	2.7	-	1.4	2.3	-
$^2J_{H(6)ax,H(6)eq}$	-	-	13.0	13.8	13.6	13.7	13.8	13.6
$^2J_{H(7)ax,H(7)eq}$	-	-	13.1	13.3	13.1	13.1	13.2	13.2
$^2J_{H(8)ax,H(8)eq}$	-	-	13.6	13.3	13.6	13.7	13.6	13.6
$^3J_{H(6)ax,H(7)ax}$	-	-	13.3	13.4	13.7	13.3	13.4	13.8
$^3J_{H(6)ax,H(7)eq}$	-	-	6.1	6.0	6.8	6.2	6.3	6.8
$^3J_{H(7)ax,H(8)ax}$	-	-	13.3	13.4	13.7	13.3	13.4	13.8
$^3J_{H(7)ax,H(8)eq}$	-	-	6.1	5.7	6.8	6.2	5.6	6.8

Table S2. ^{13}C NMR data: chemical shift (δ) and ^{1}JC,H (Hz) for compounds **1–8** in $CDCl_3$

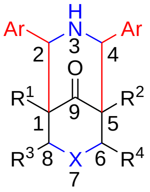

Comp.	X	Ar	R1	R2	R3	R4
1	NH	C_6H_5	H	H	C_6H_5	C_6H_5
2	NH	C_6H_5	H	CH_3	C_6H_5	C_6H_5
3	CH_2	C_6H_5	H	H	H	H
4	CH_2	C_6H_5	H	CH_3	H	H
5	CH_2	C_6H_5	CH_3	CH_3	H	H
6	CH_2	3NO_2-C_6H_4	H	H	H	H
7	CH_2	3NO_2-C_6H_4	H	CH_3	H	H
8	CH_2	3NO_2-C_6H_4	CH_3	CH_3	H	H

	1	2	3	4	5	6	7	8
C(1)	63.41	54.84	54.1	50.94	51.11	53.29	50.55	51.06
C(2)	58.77	61.64	64.87	71.33	71.65	64.02	70.42	70.81
C(4)	58.77	58.64	64.87	65.06	71.65	64.02	64.21	71.20
C(5)	63.41	62.57	54.1	54.49	51.11	53.29	53.68	50.56
C(6)	61.80	63.31	29.17	29.23	37.02	28.92	29.01	36.98
C(7)	-	-	21.24	21.54	21.58	21.18	21.45	21.48
C(8)	61.80	70.30	29.17	36.96	37.02	28.92	36.74	36.94
C(9)	211.66	212.70	217.74	218.01	218.16	214.92	215.46	215.56
Me	-	18.41	-	20.43	20.93	-	20.25	20.73
$^{1}J_{C(1),Heq}$	138.7	-	136.3	-	-	136.3	-	-
$^{1}J_{C(2),Hax}$	140.5	138.4	134.4	133.9	134.0	137.4	135.9	136.0
$^{1}J_{C(4),Hax}$	140.5	138.7	134.4	135.0	134.0	137.4	135.9	135.9
$^{1}J_{C(5),Heq}$	138.7	138.0	136.3	136.3	-	136.3	136.2	-
$^{1}J_{C(6),Hax}$	140.6	136.4	125.9	128.1	125.3	133.4	127.6	132.2
$^{1}J_{C(6),Heq}$	-	-	130.4	131.8	130.5	133.5	130.2	131.4
$^{1}J_{C(7),Hax}$	-	-	131.5	131.7	130.9	130.2	130.0	130.5
$^{1}J_{C(7),Heq}$	-	-	125.9	125.9	125.2	125.5	126.4	125.4
$^{1}J_{C(8),Hax}$	140.6	136.4	125.9	124.4	125.3	125.4	126.8	125.5
$^{1}J_{C(8),Heq}$	-	-	130.4	130.2	130.5	133.5	131.7	133.3
Me	-	128.5	-	126.7	126.6	-	127.3	127.5

Schemes:

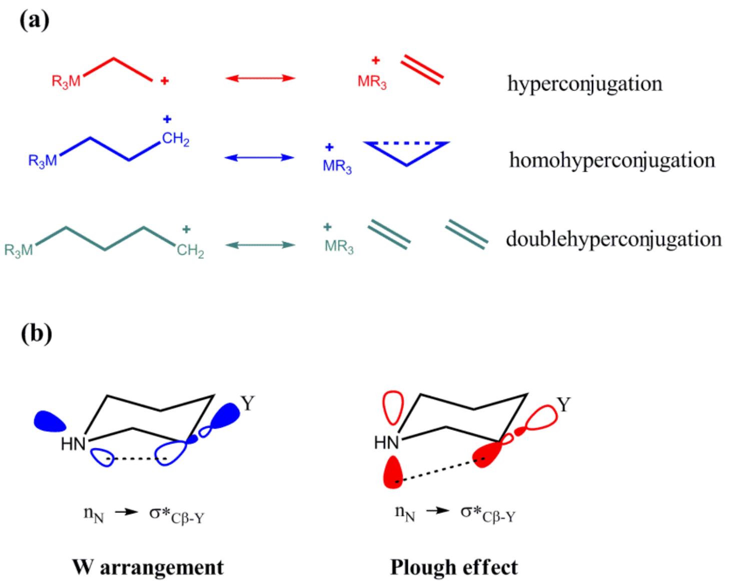

Scheme S1. (a) Stabilization of positive charges by the homohyperconjugation effect in organometallic molecules. (b) Stabilization of conformation by LPE (N, O and S) homohyperconjugation through W arrangement and Plough effect.

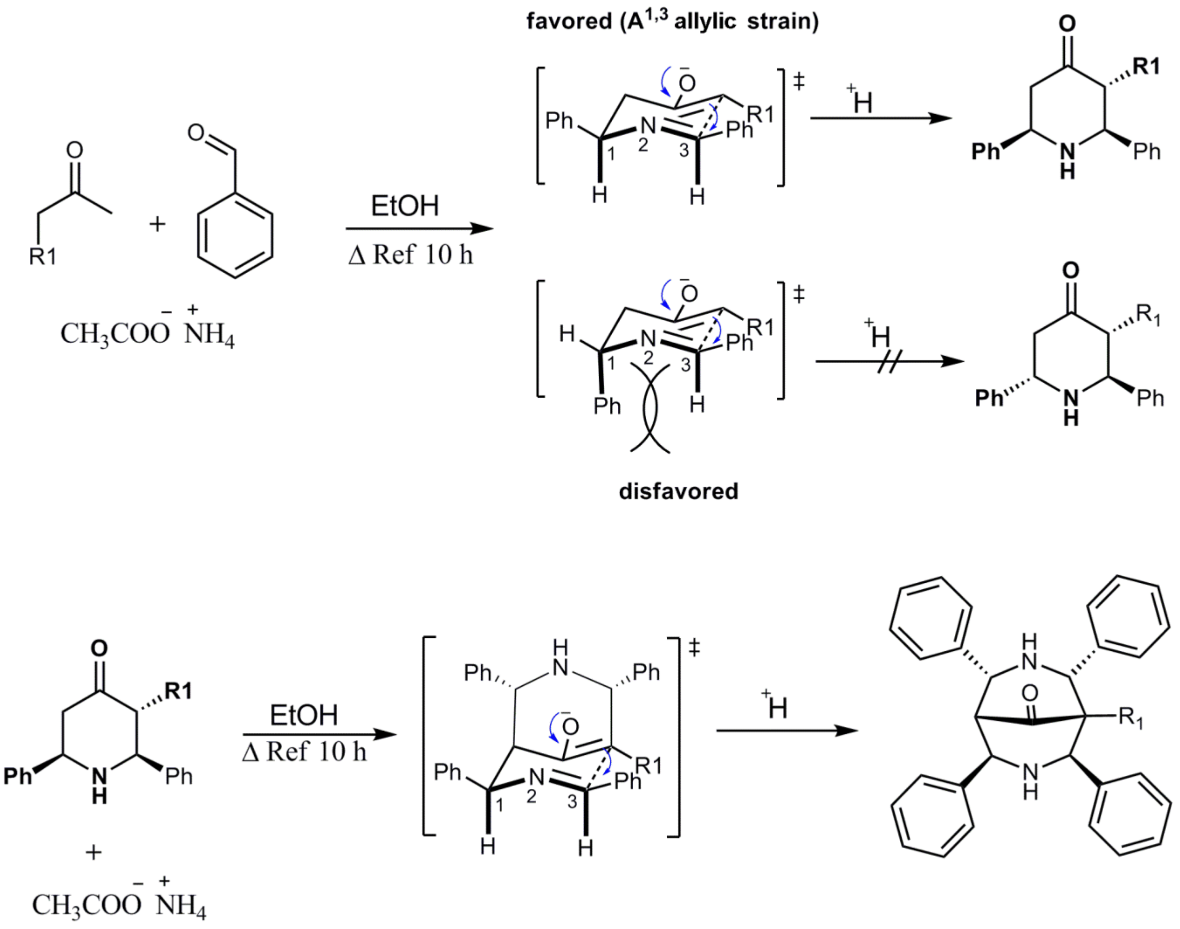

Scheme S2. Reaction mechanism proposed for the synthesis of piperidones **1** and **2** by double Mannich reaction.

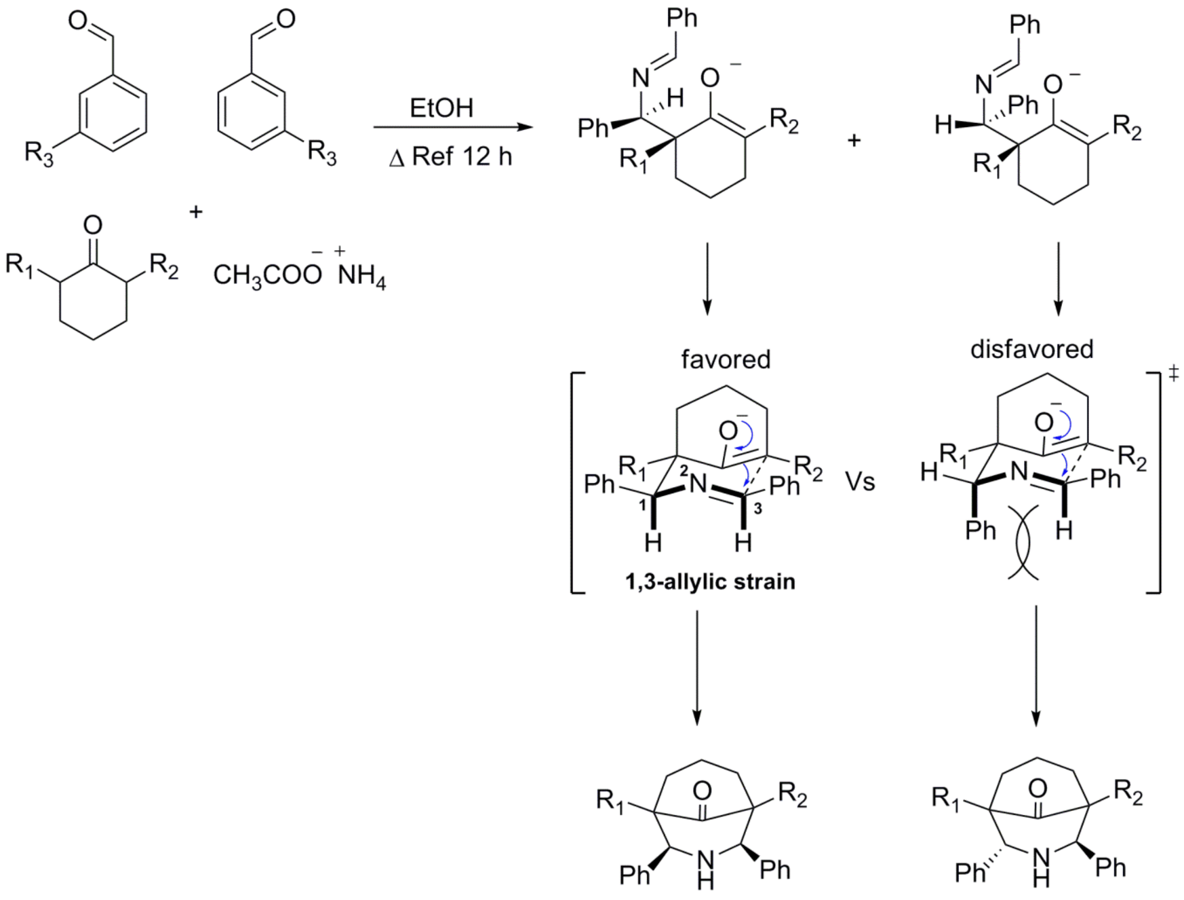

Scheme S3. Reaction mechanism proposed for the synthesis of piperidones **3** to **8** by Mannich reaction.

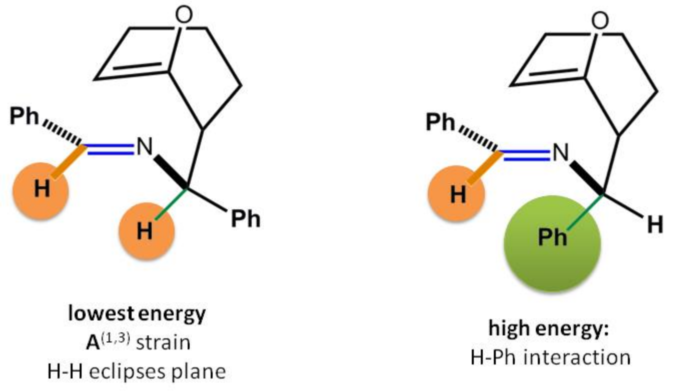

Scheme S4. A(1,3) allylic strain.

(a)

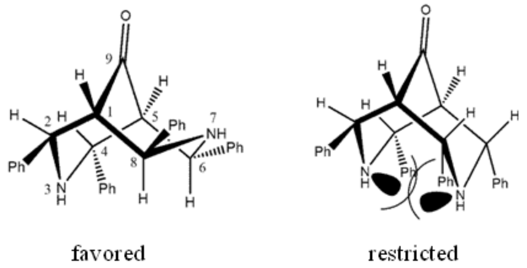

(b)

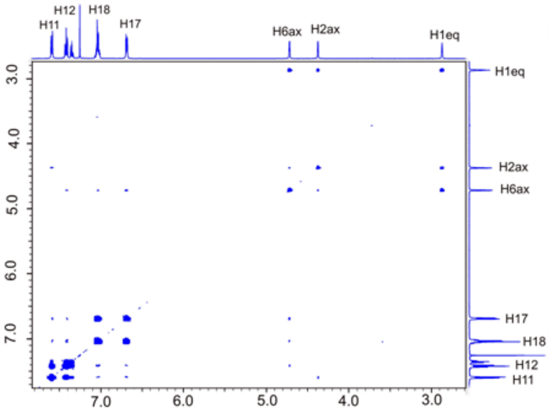

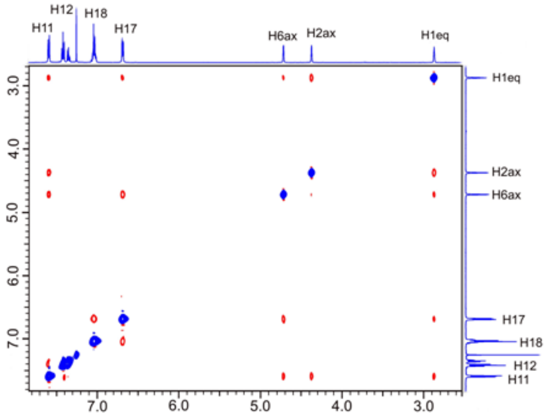

Scheme S5. (a) preferred conformation for the compound **1** and **2**. (b) dqf- COSY spectrum of compound **1** in $CDCl_3$. (c) t-ROESY spectrum of compound **1** in $CDCl_3$.

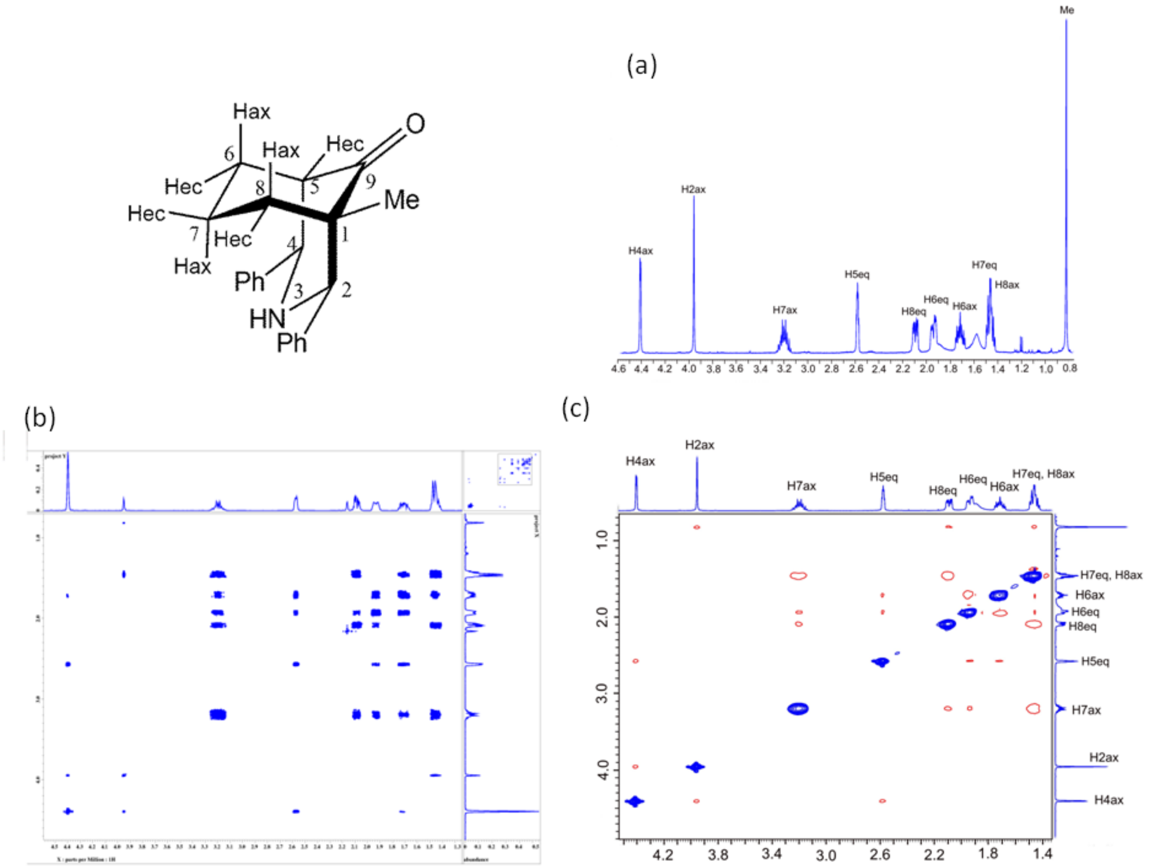

Scheme S6. NMR characterization of compound **4** in $CDCl_3$. (a) ^1H spectrum. (b) dqf-COSY spectrum. (c) HSQC spectrum.

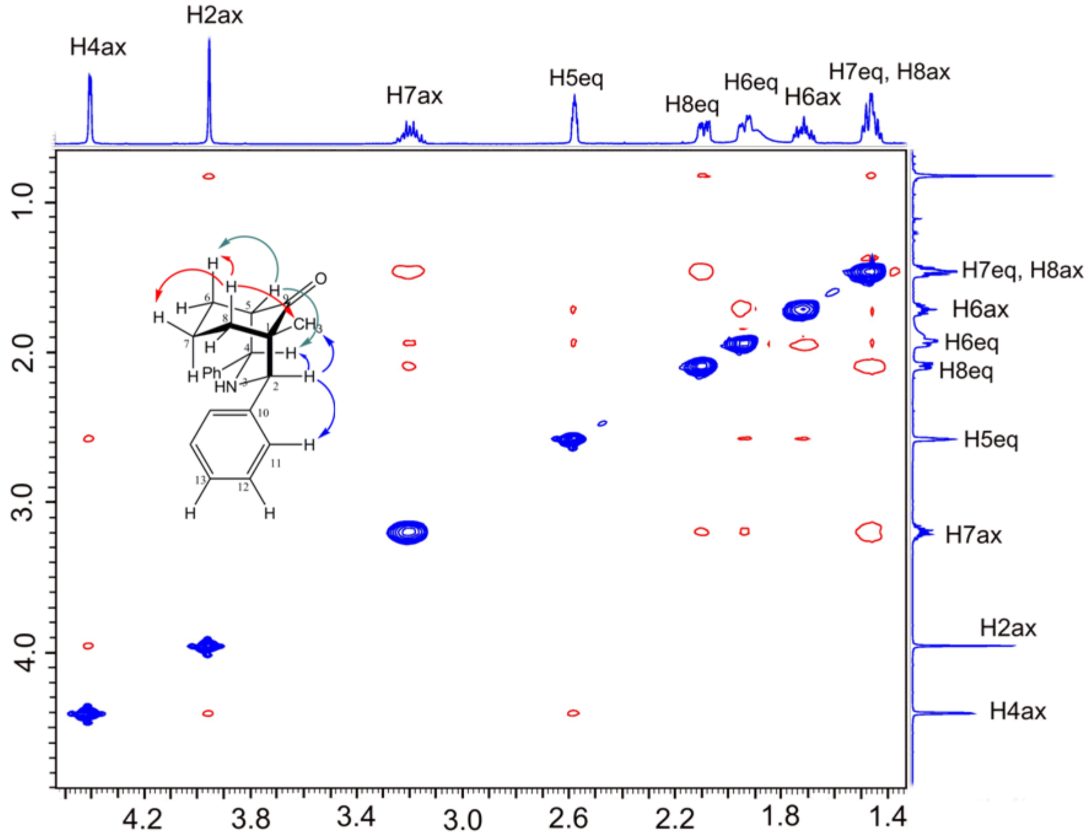

Scheme S7. Determination of the conformation of piperidones by t-ROESY spectrum

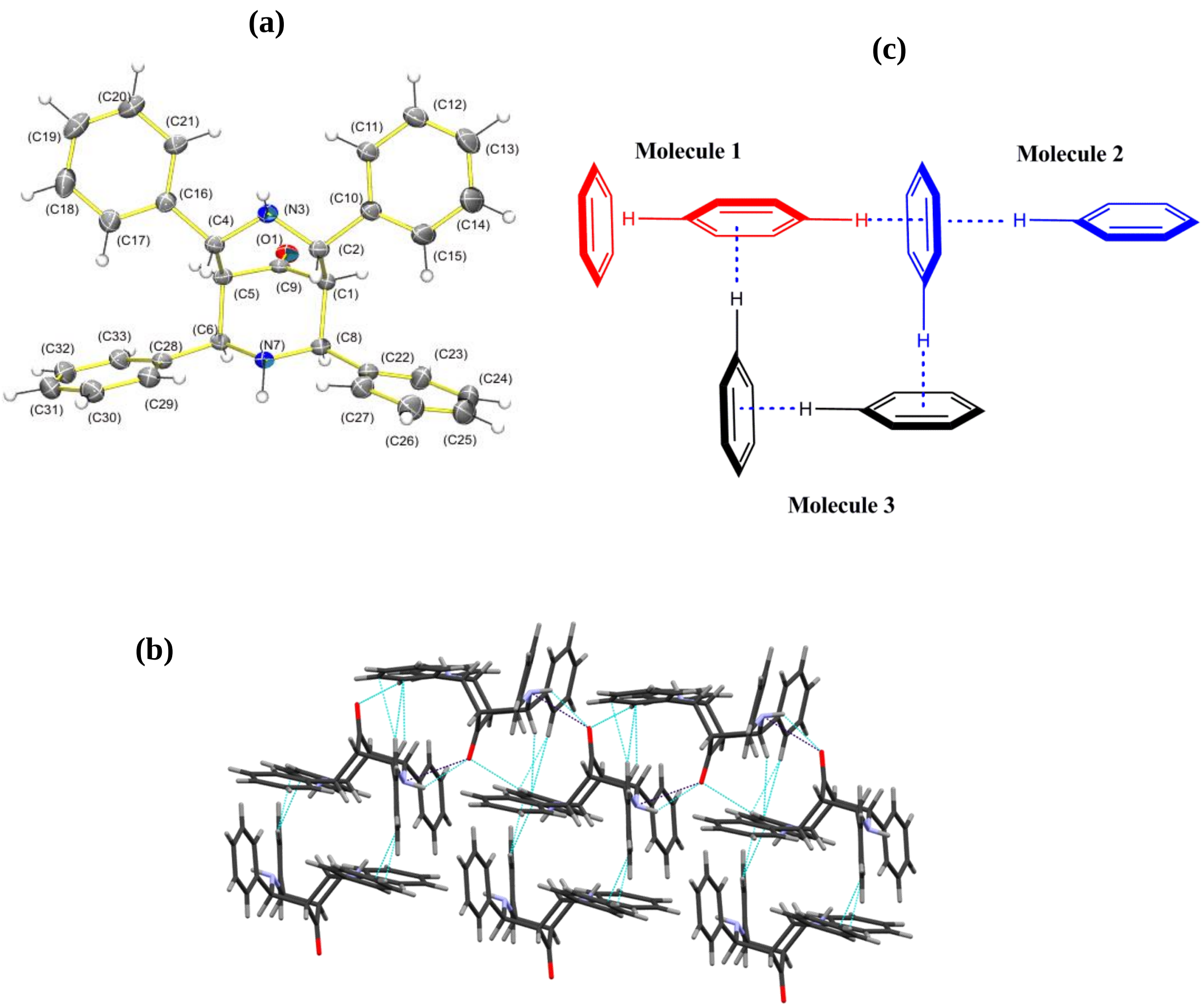

Figure S8. (a) ORTEP diagram of compounds **1**, thermal ellipsoids are drawn at 30% probability level for all atoms other than H. **(b)** crystal packing and C(15)···H(15)···π inter and intramolecular interaction of compound **1**.

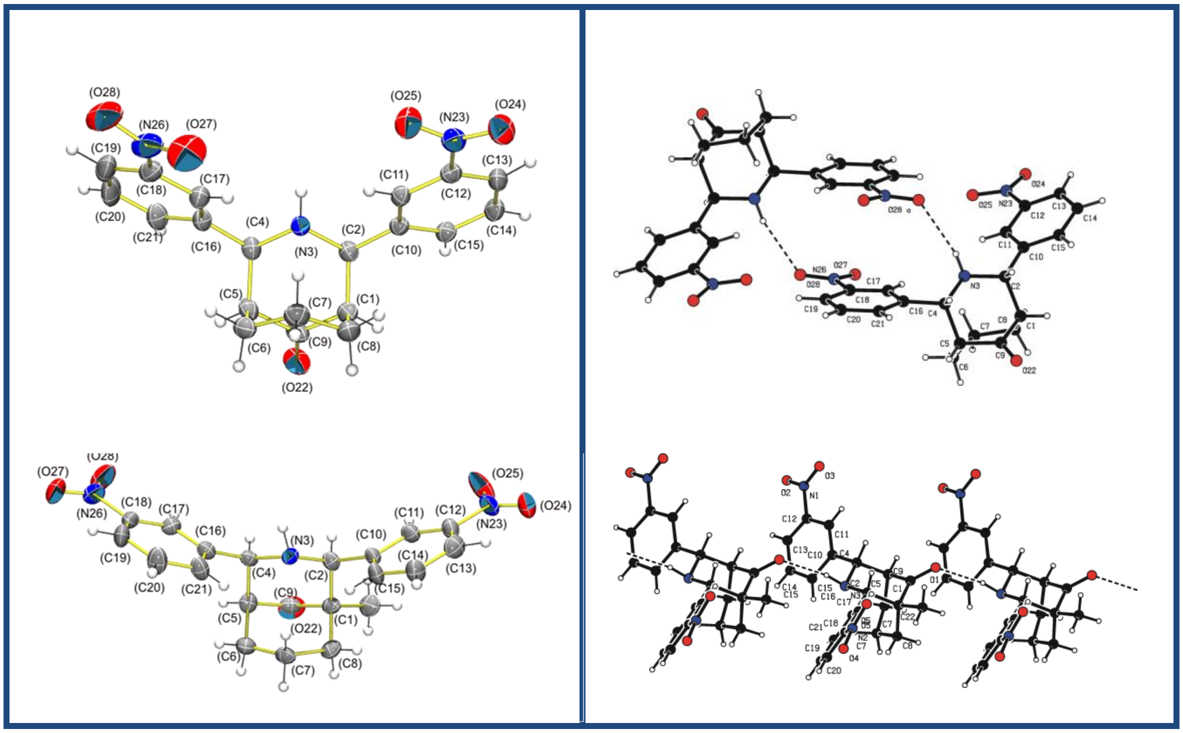

Scheme S9. (a) ORTEP diagram of compounds **6** and **7**, thermal ellipsoids are drawn at 30% probability level for all atoms other than H. **(b)** Intermolecular hydrogen bonds of compound **6** and **7.**

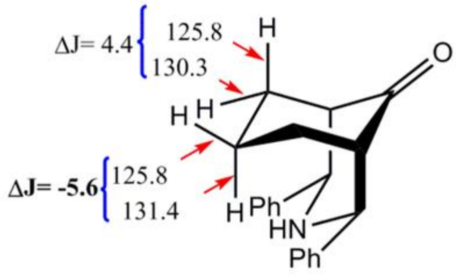

3

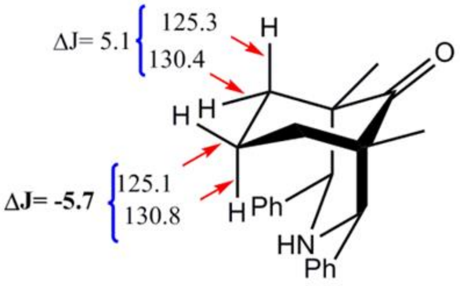

5

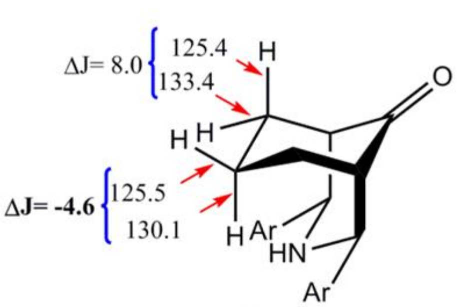

6

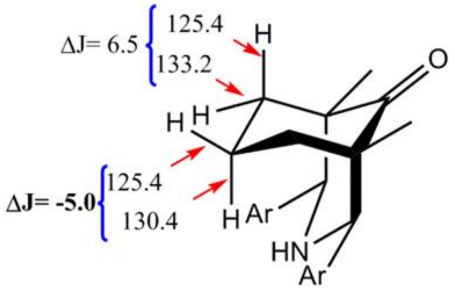

8

Scheme S10. $^{1}J_{C,H}$ coupling constant of compound **3**, **5**, **6** and **8**

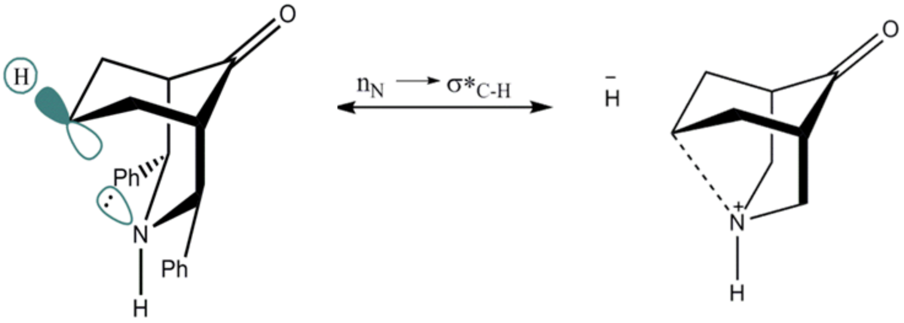

Scheme S11. Schematic representation of $n_X \rightarrow \sigma^*_{C7\text{-}H_{eq}}$ Hyperconjugation in the piperidones **3-8**.

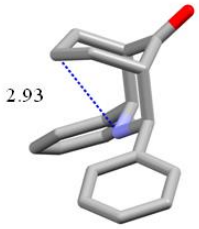

3

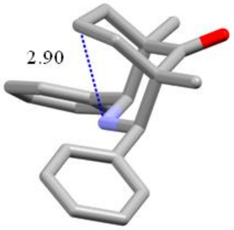

5

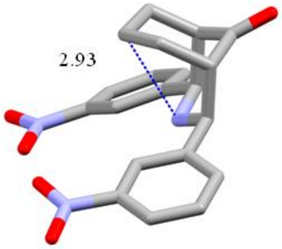

6

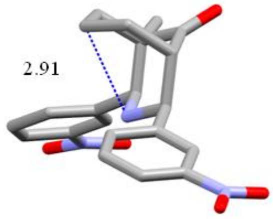

7

Scheme S12. Distance between N(3) and σ*C-H orbital of compounds **3**, **5**, **6** and **7,** measured by X-ray

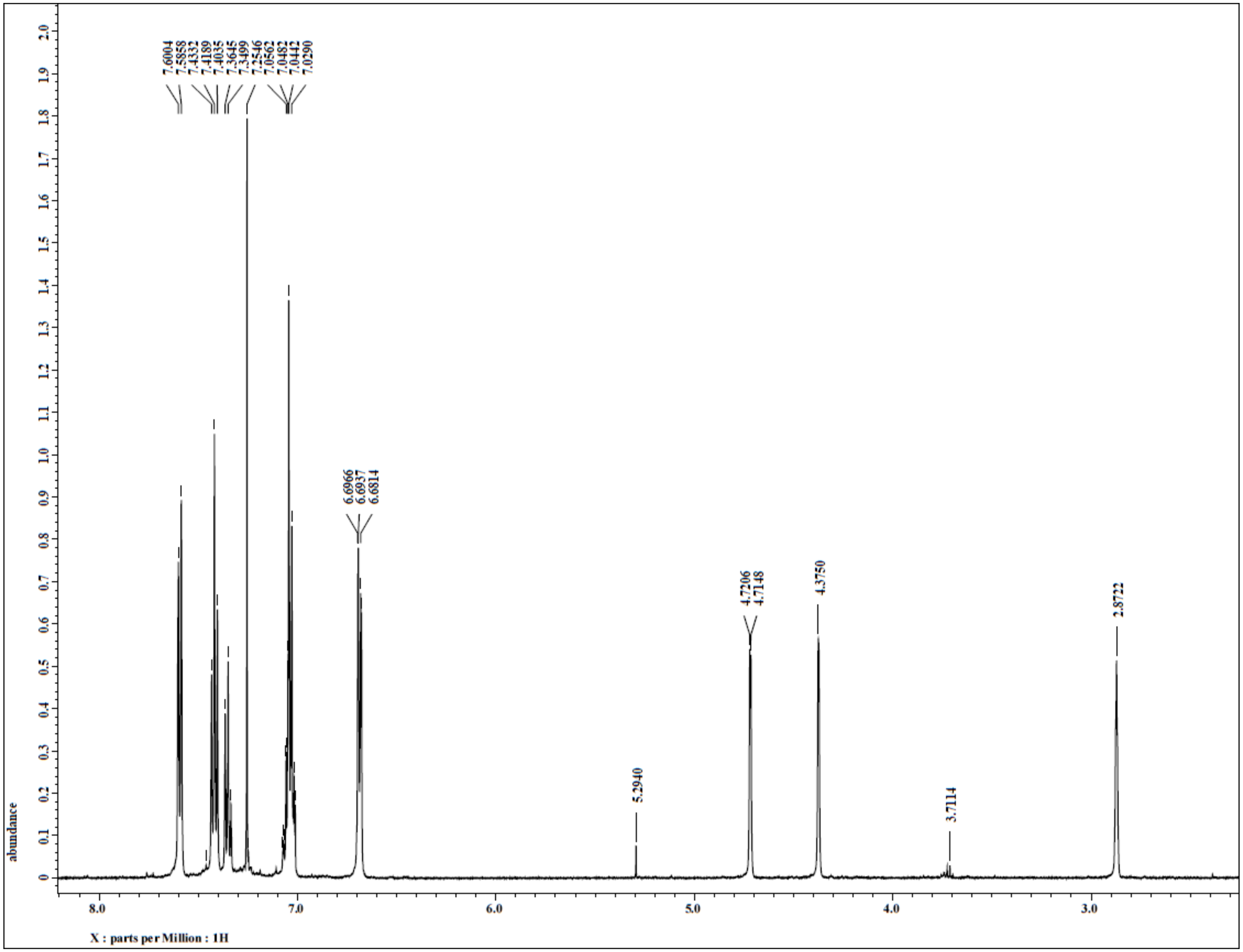

^{1}H NMR spectrum of **1**

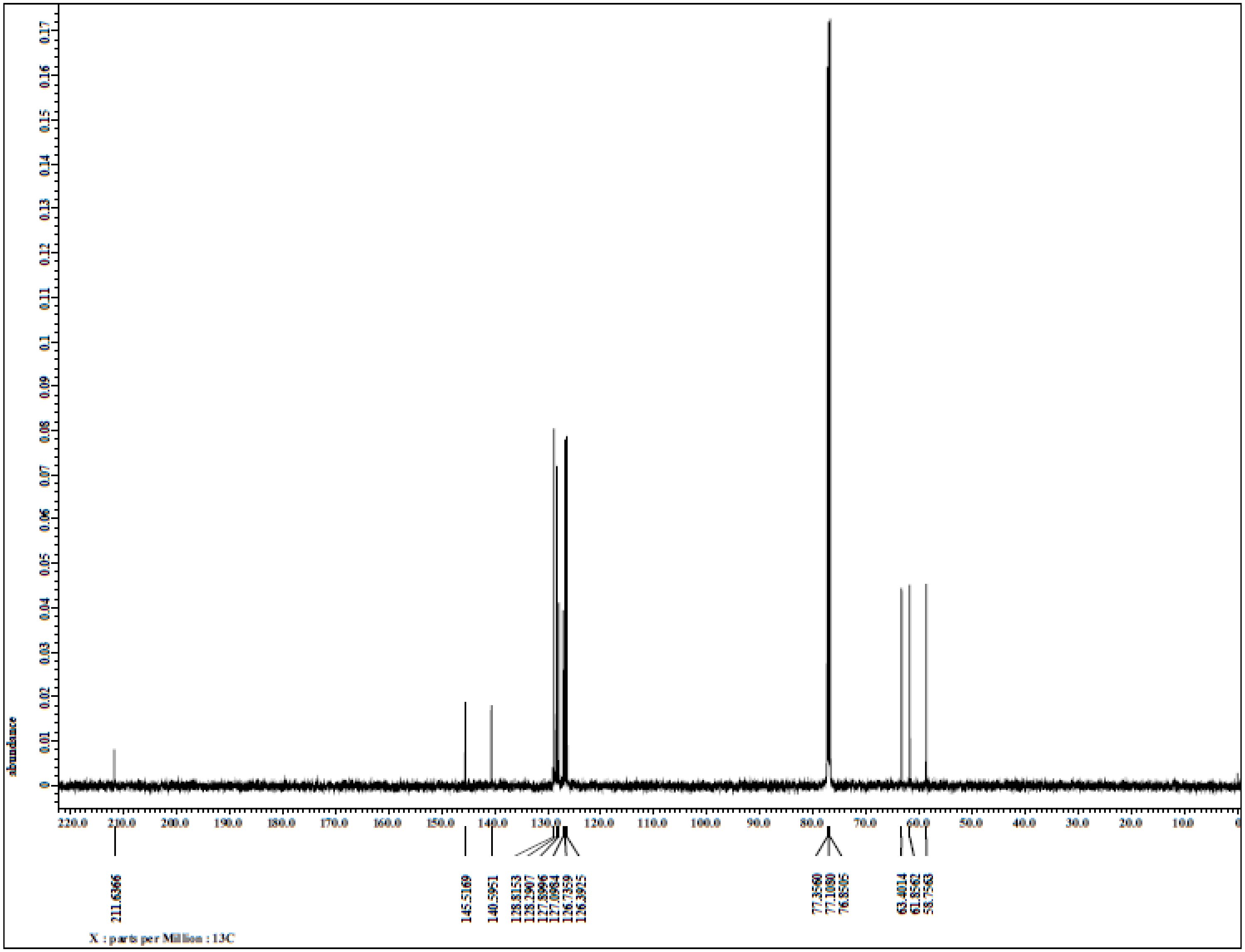

^{13}C NMR spectrum of **1**

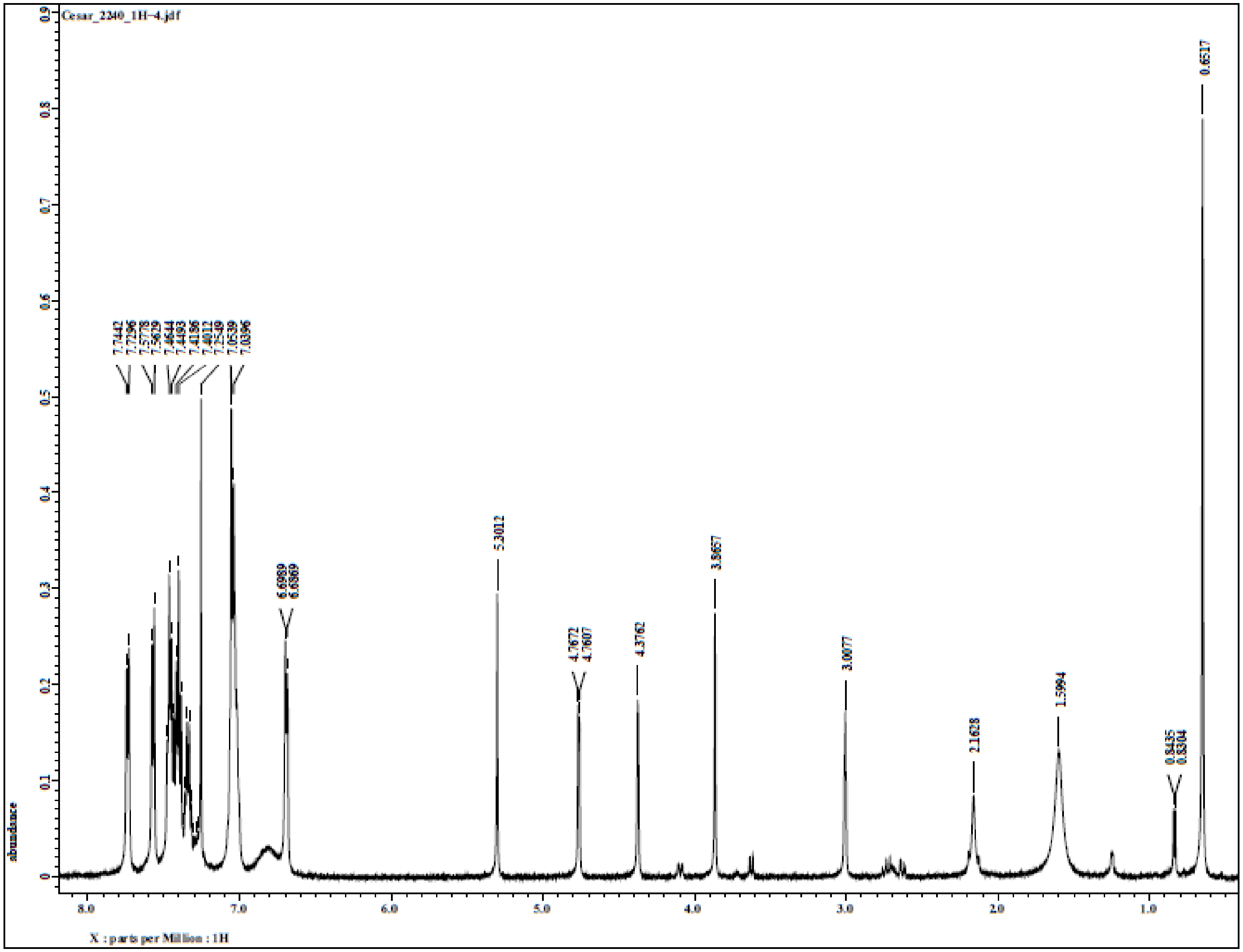

^{1}H NMR spectrum of **2**

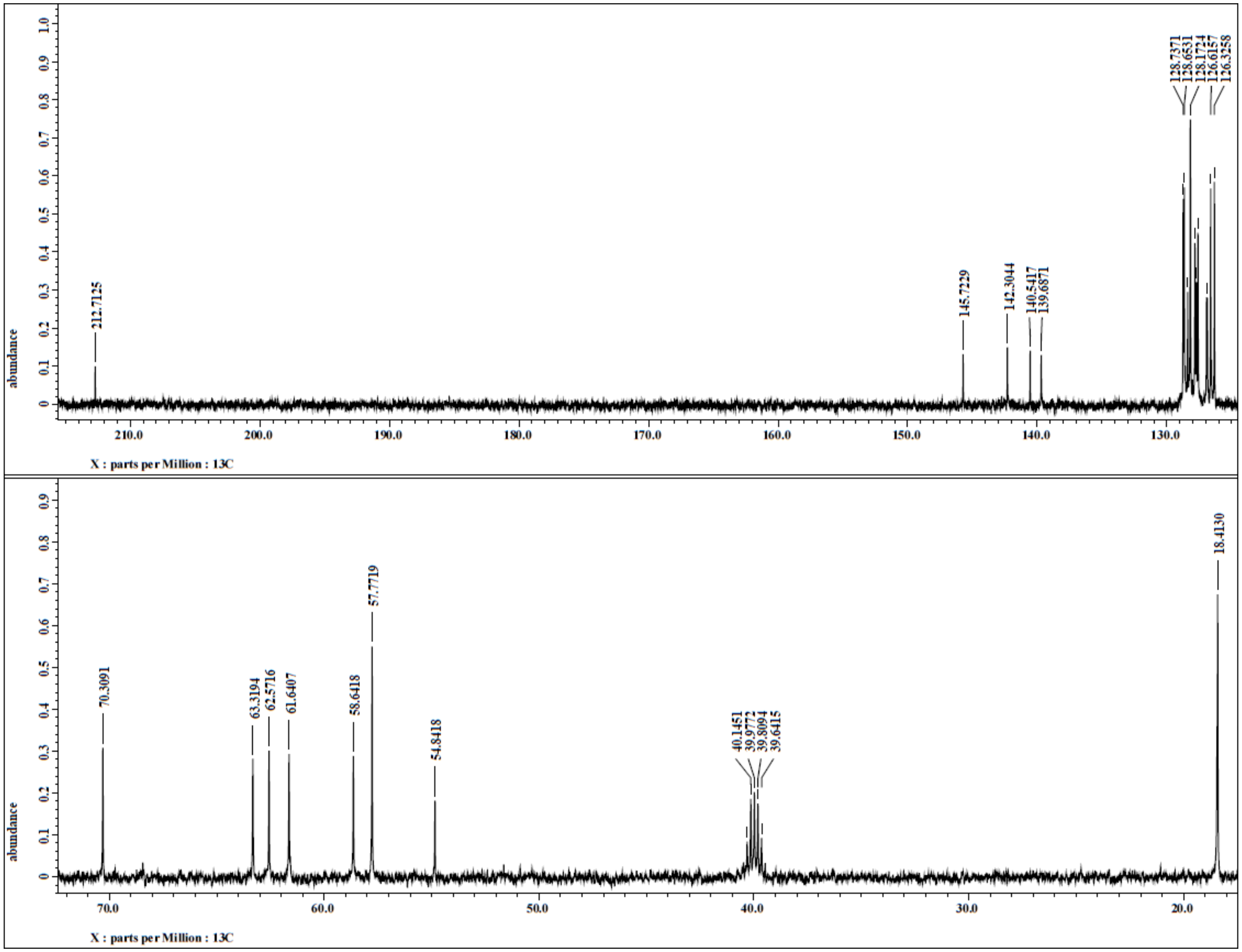

^{13}C NMR spectrum of **2**

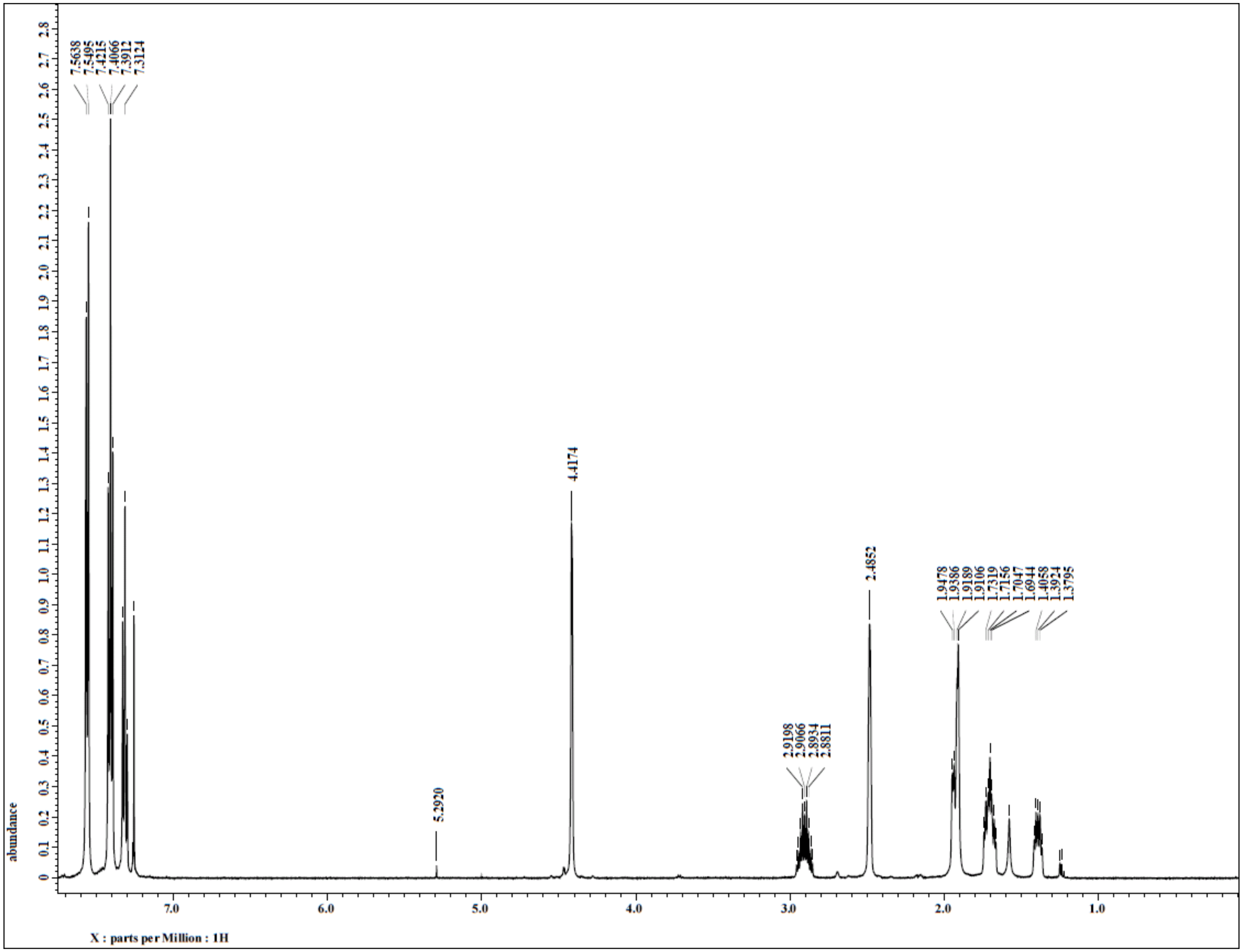

^{1}H spectrum of compound **3**

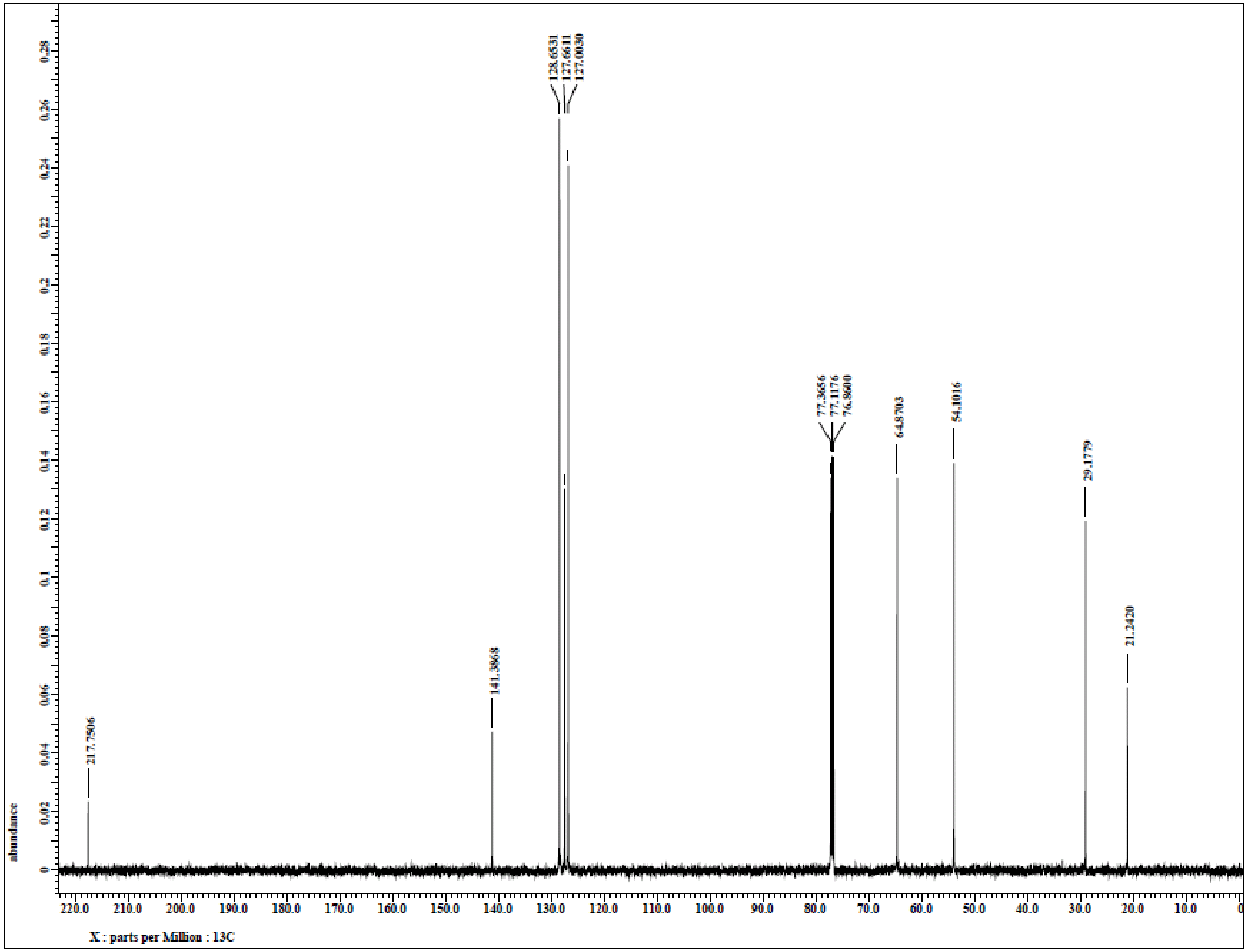

^{13}C spectrum of compound **3**

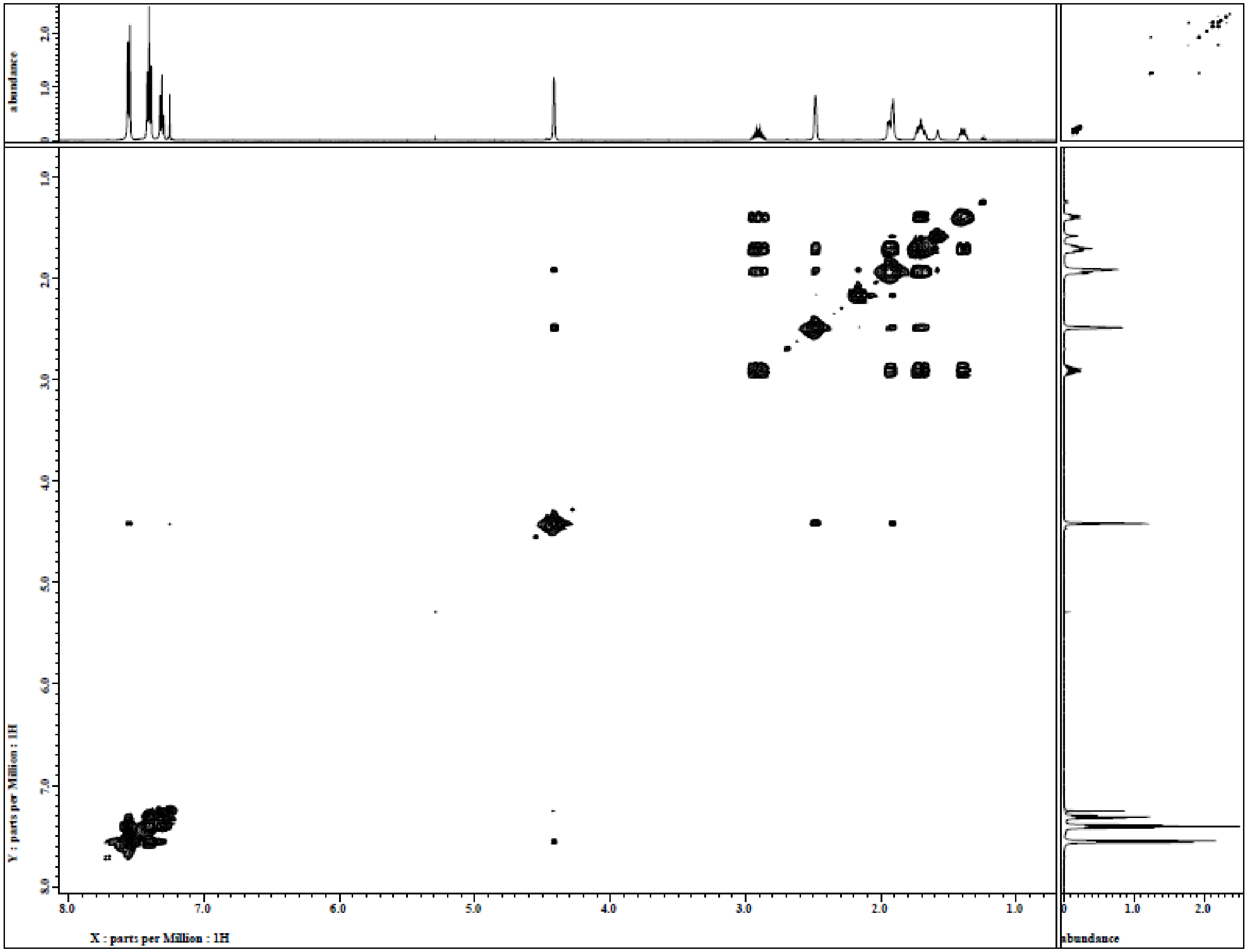

^{1}H-^{1}H COSY spectrum of compound **3**

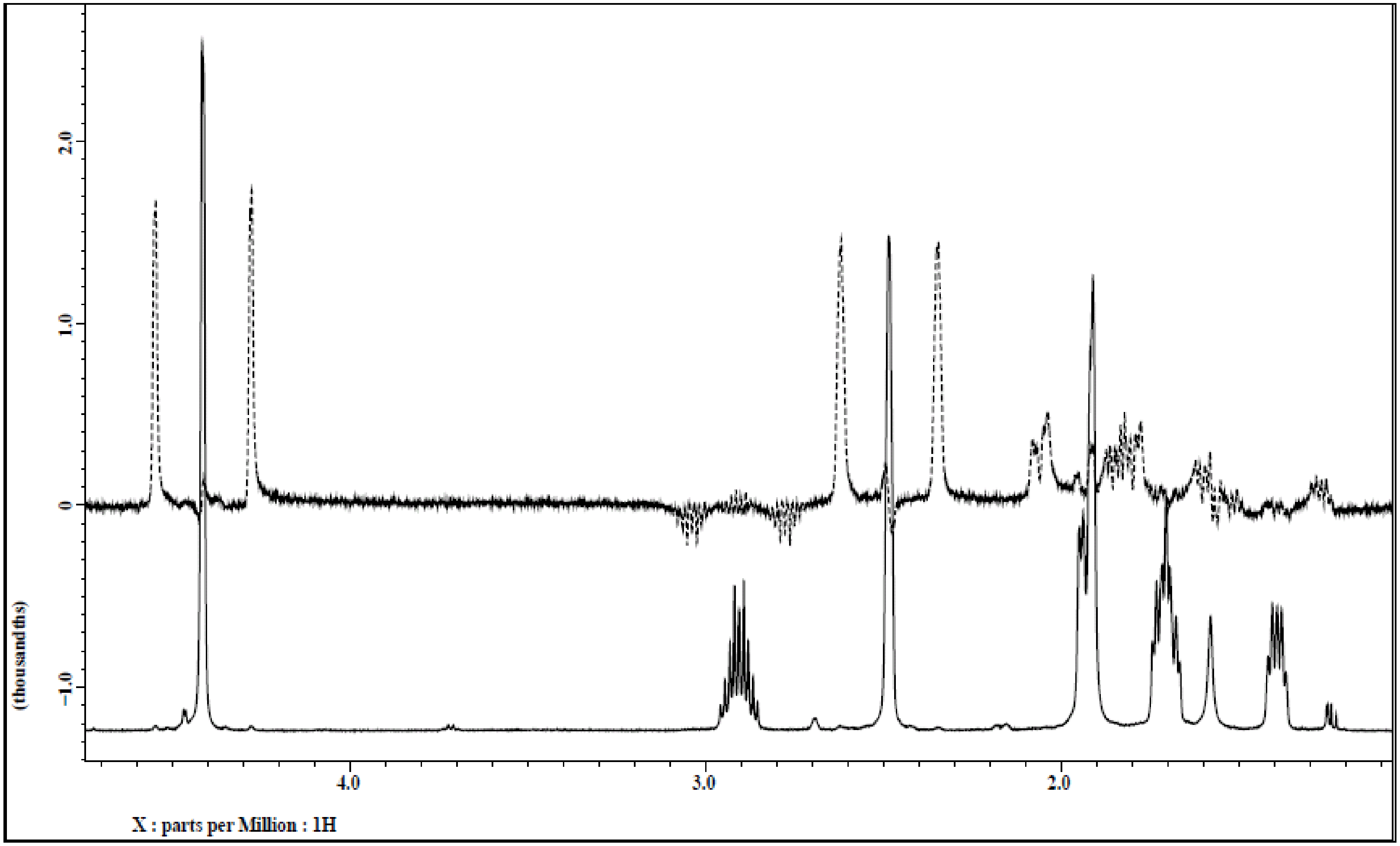

DQF spectrum of compound **3**

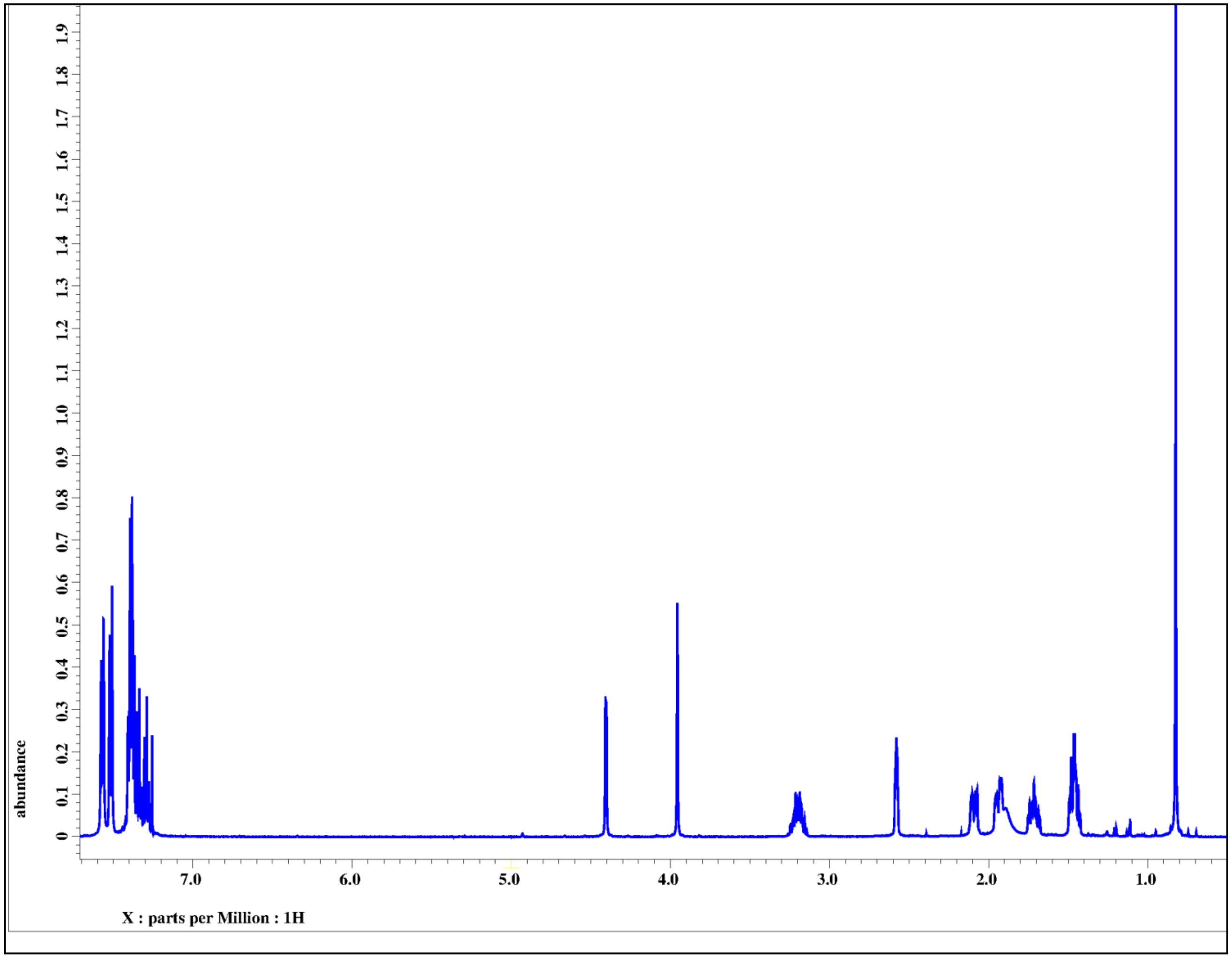

^{1}H NMR spectrum of compound **4**

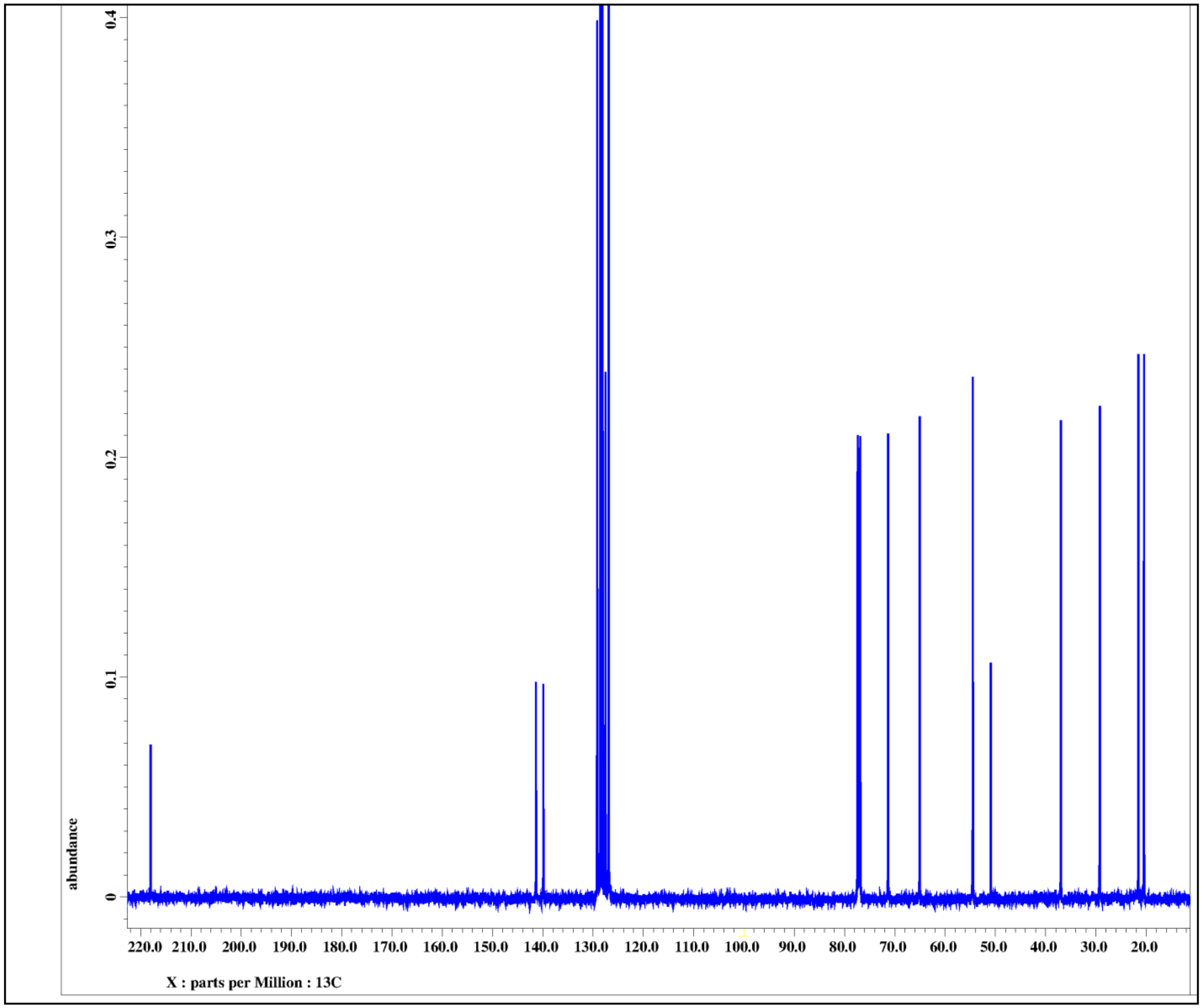

^{13}C NMR spectrum of compound **4**

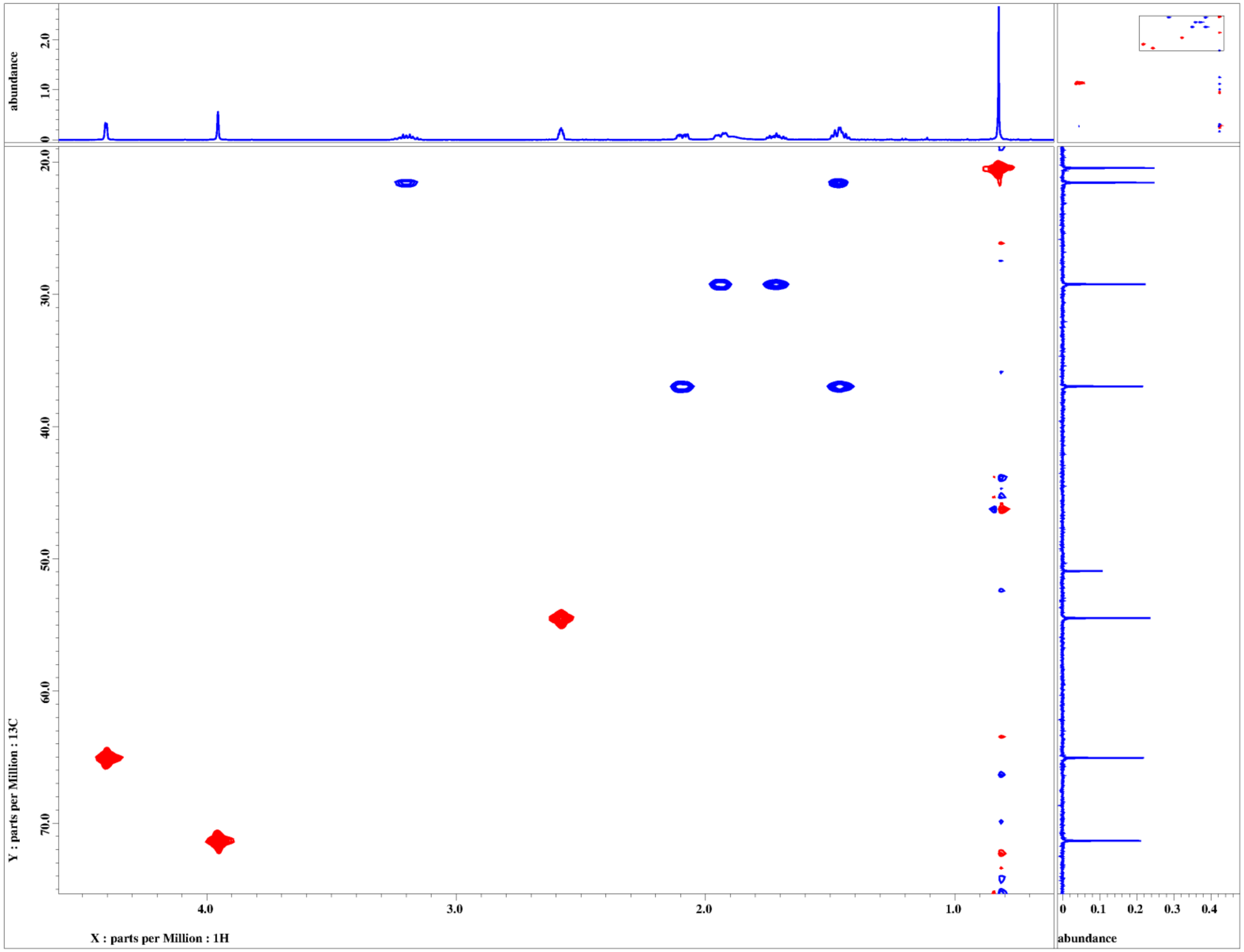

HSQC spectrum of compound **4**

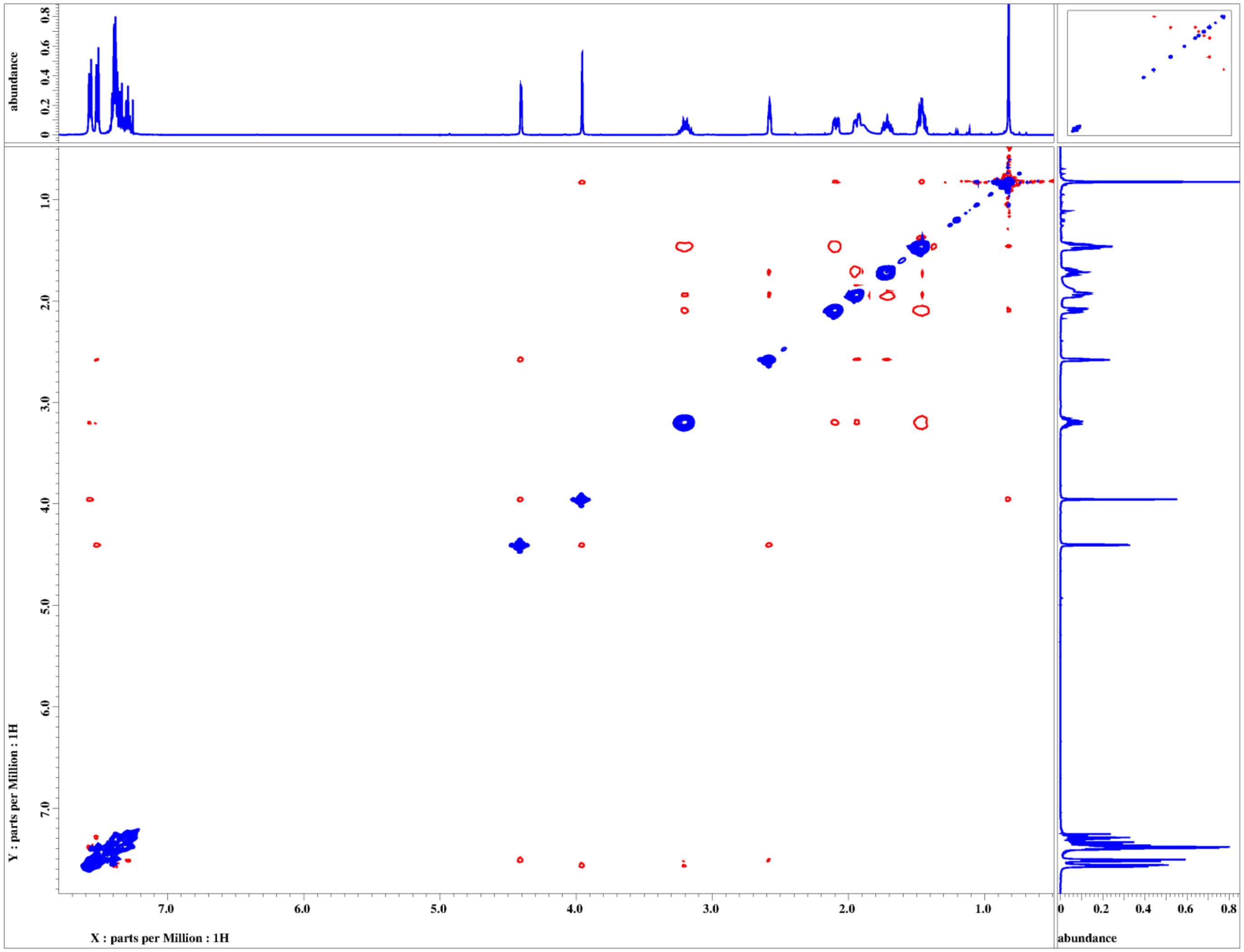

t-ROESY spectrum of compound **4**

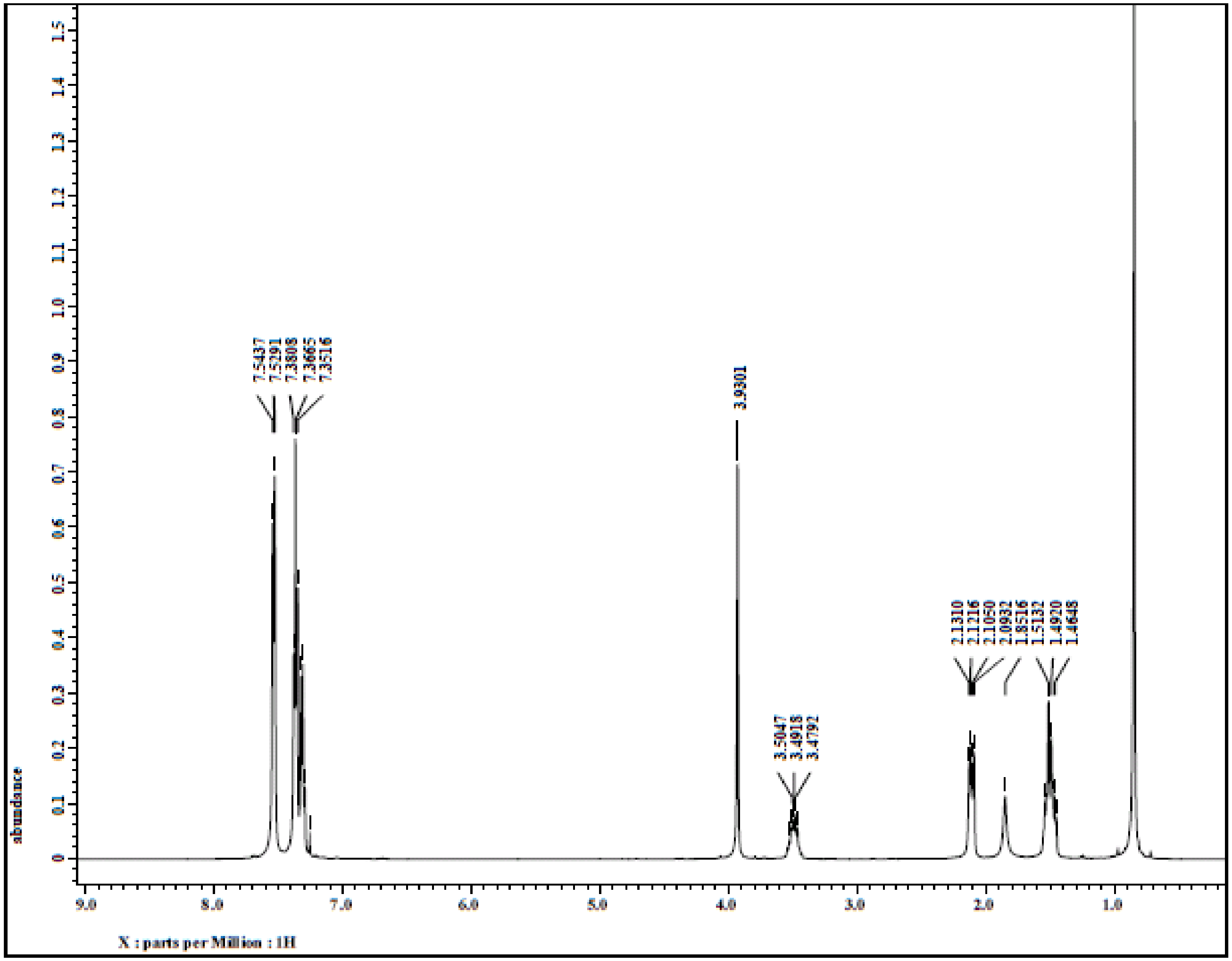

^{1}H spectrum of compound **5**

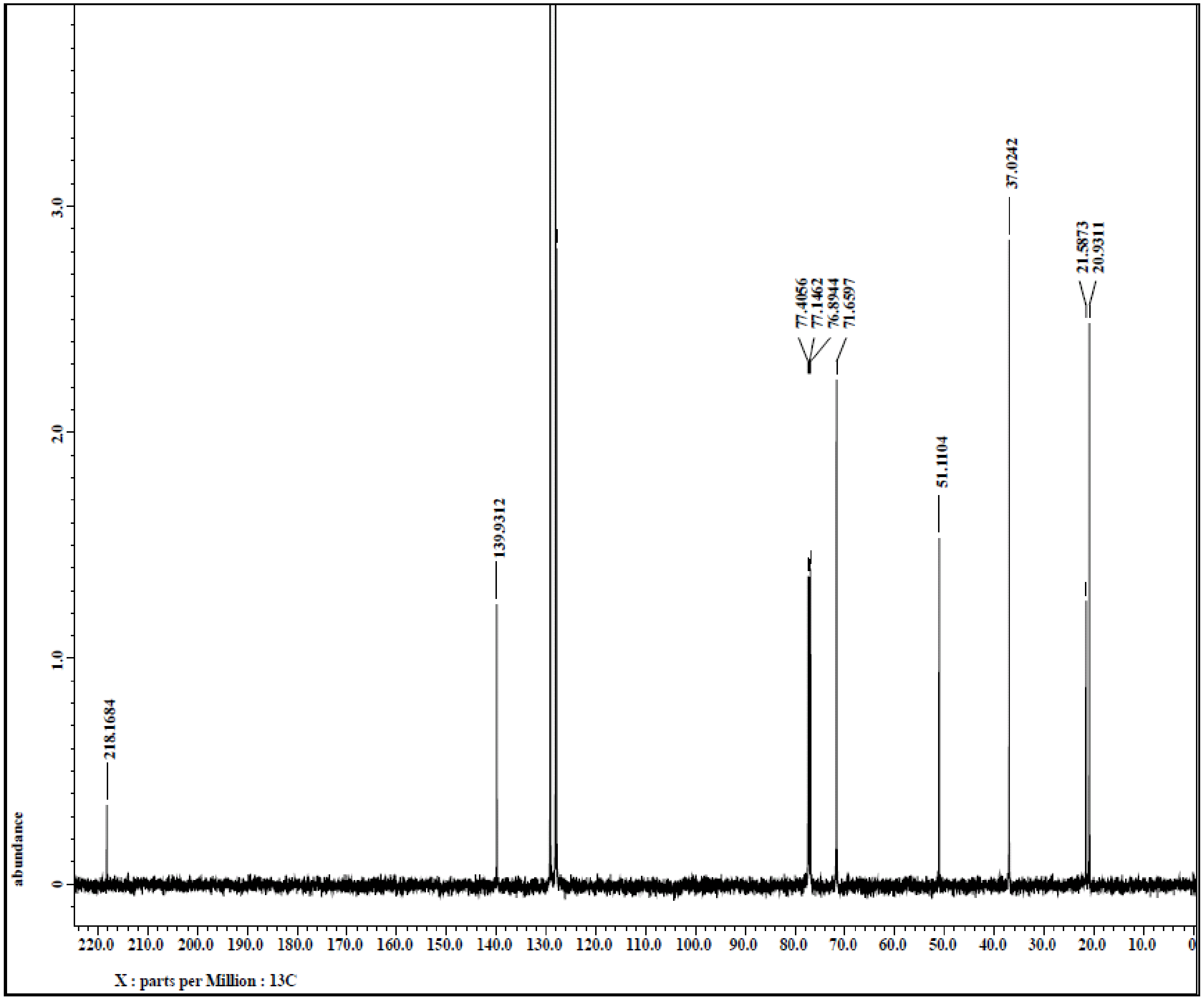

^{13}C spectrum of compound **5**

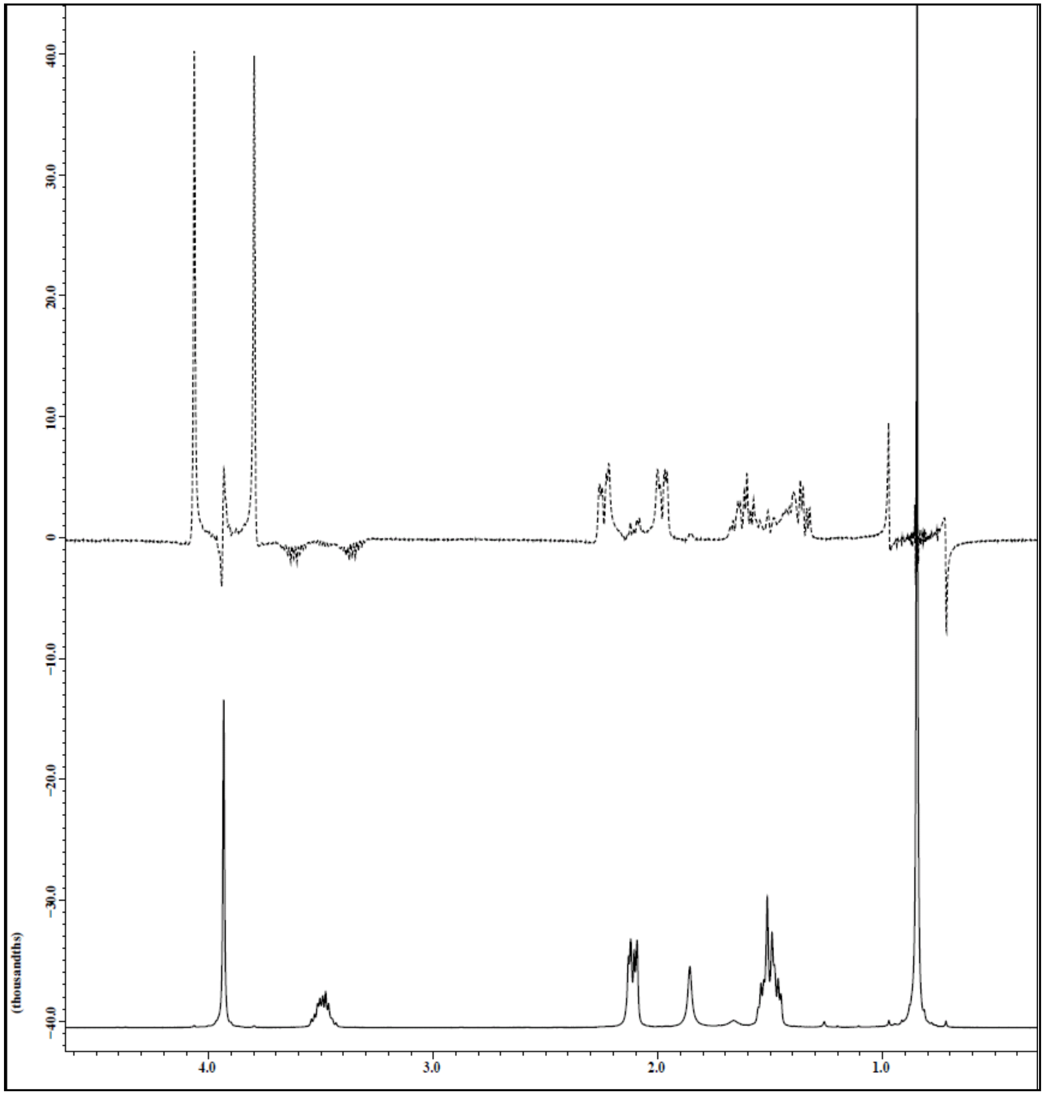

dqf spectrum of compound **5**

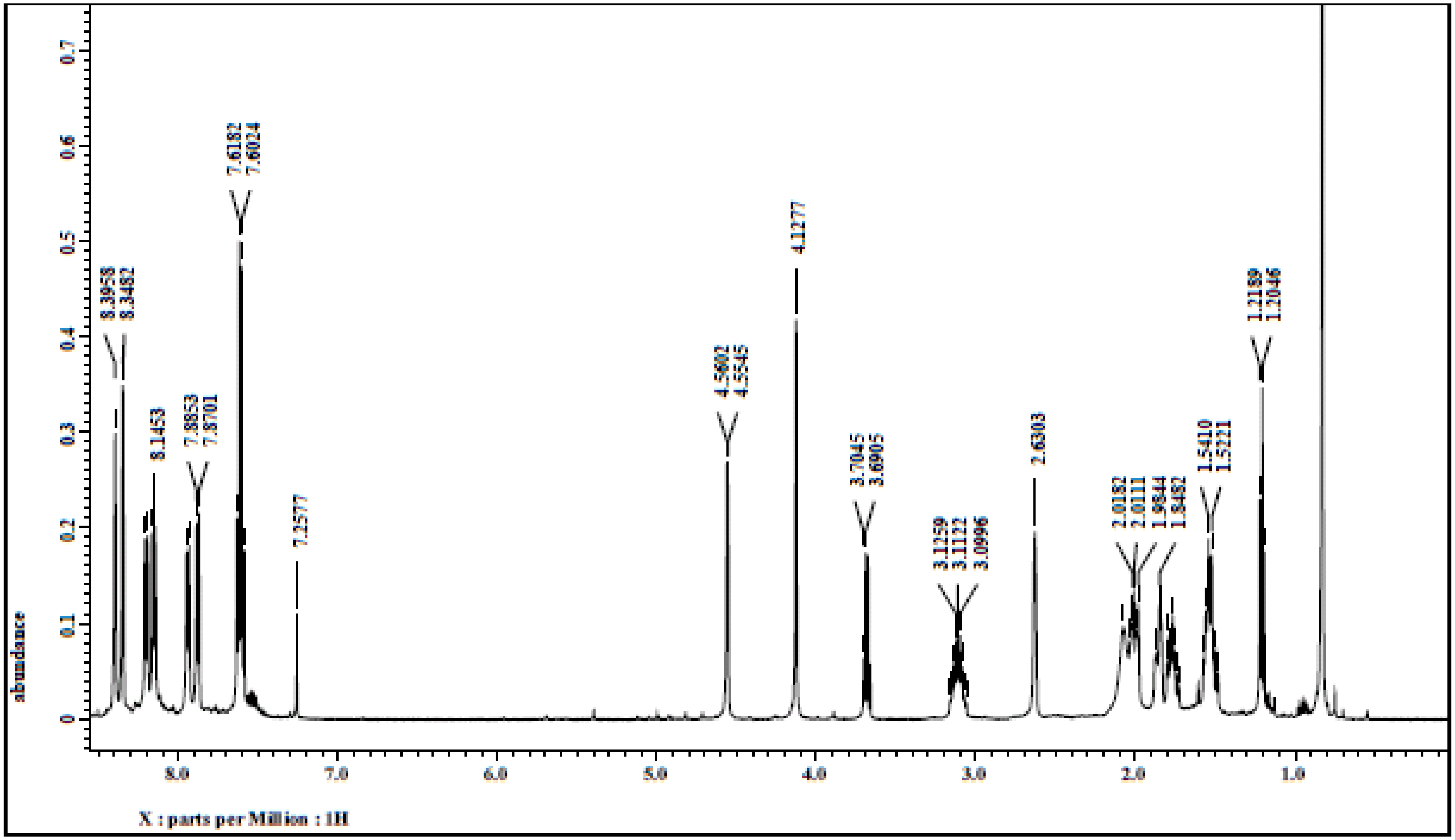

^{1}H spectrum of compound **6**

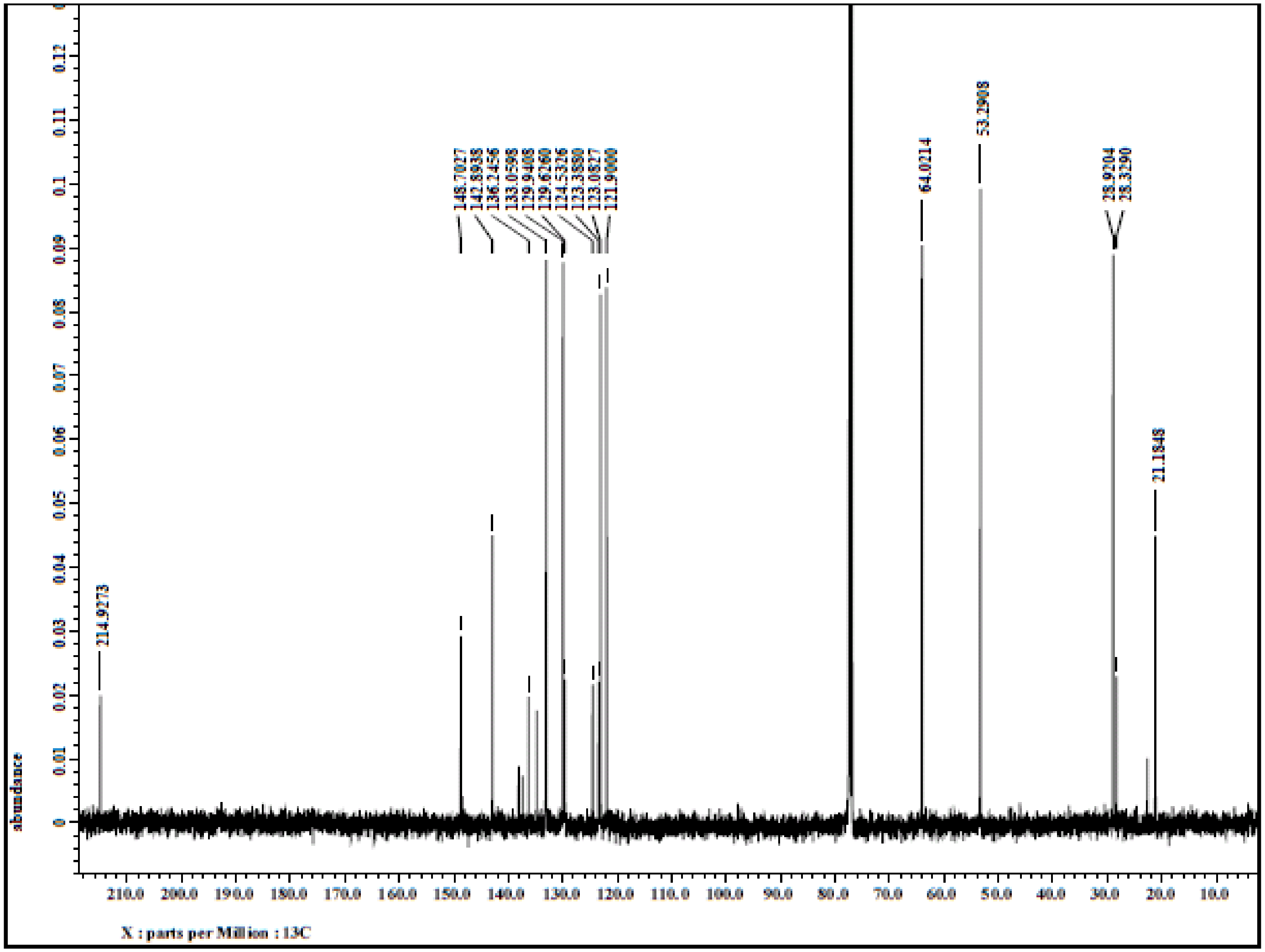

^{13}C spectrum of compound **6**

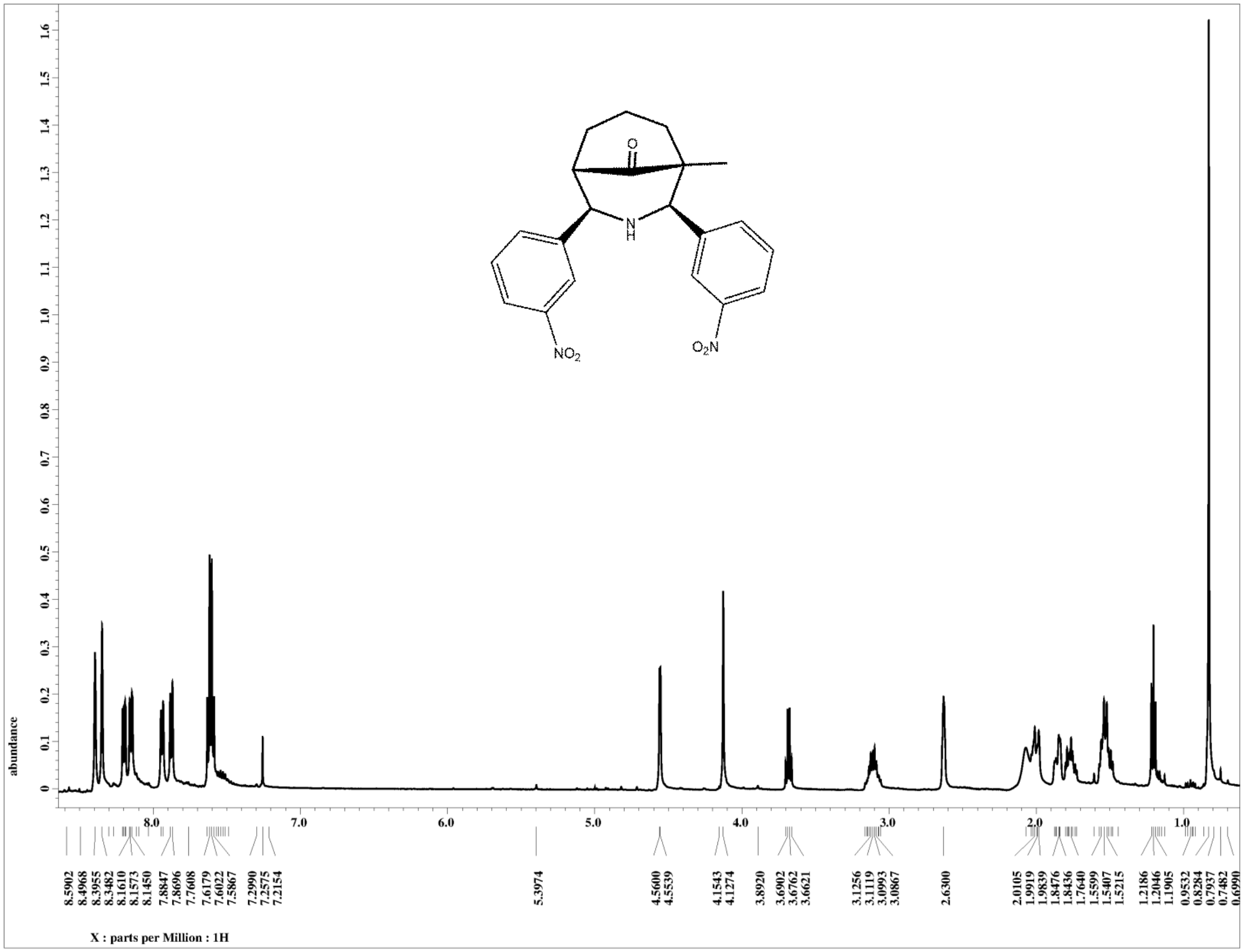

^{1}H NMR spectrum of compound **7**

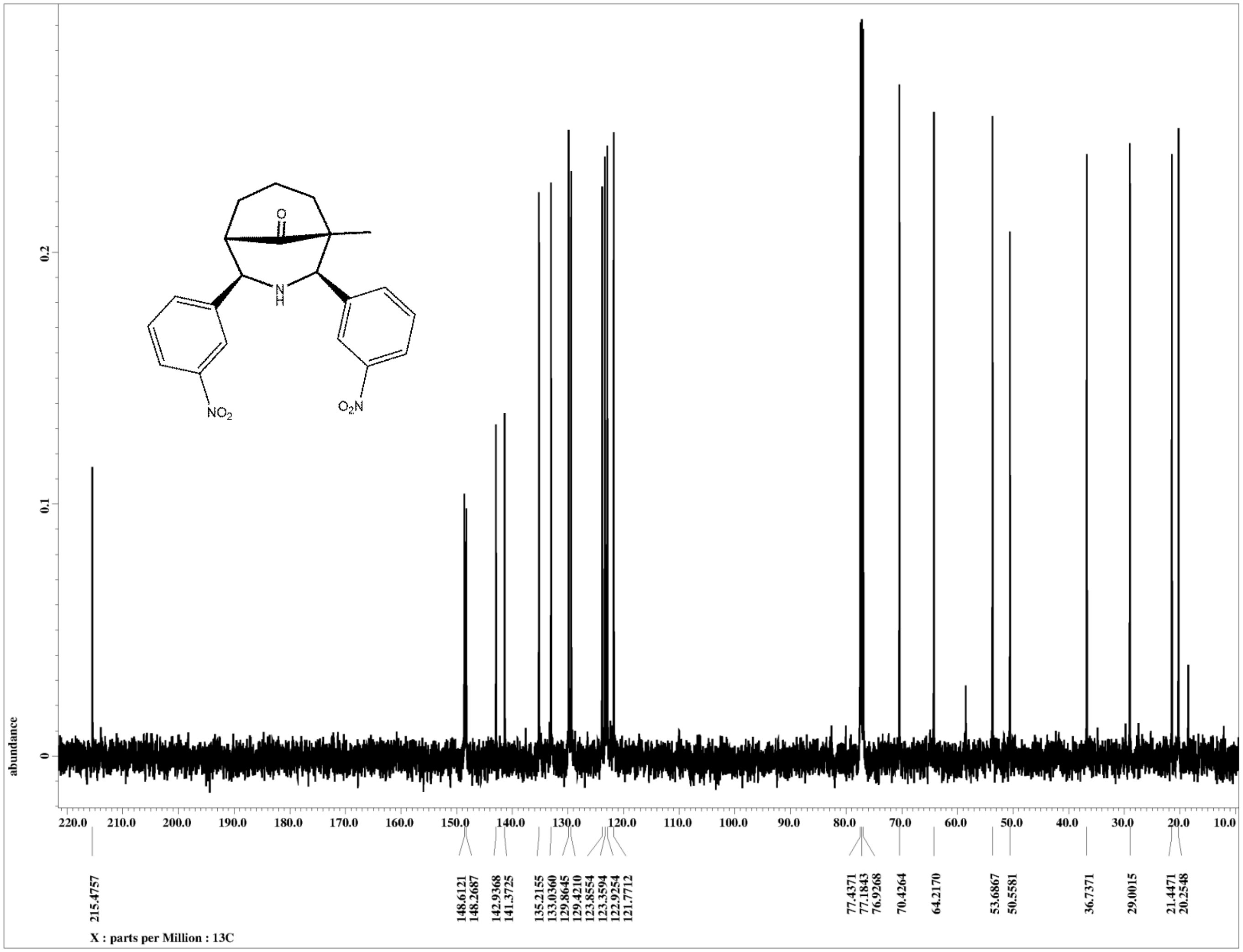

^{13}C NMR spectrum of compound **7**

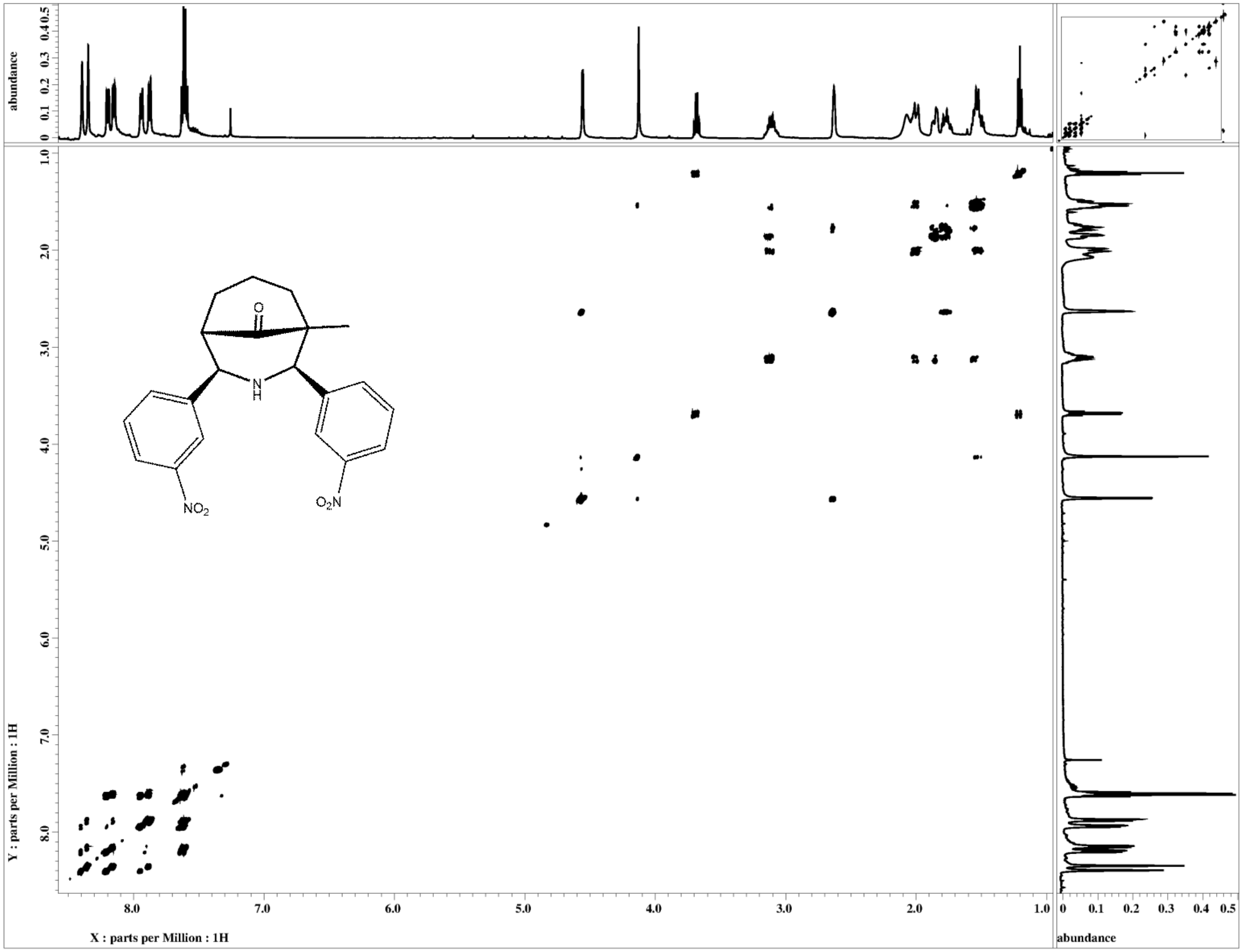

^1H-^1H COSY spectrum of compound **7**

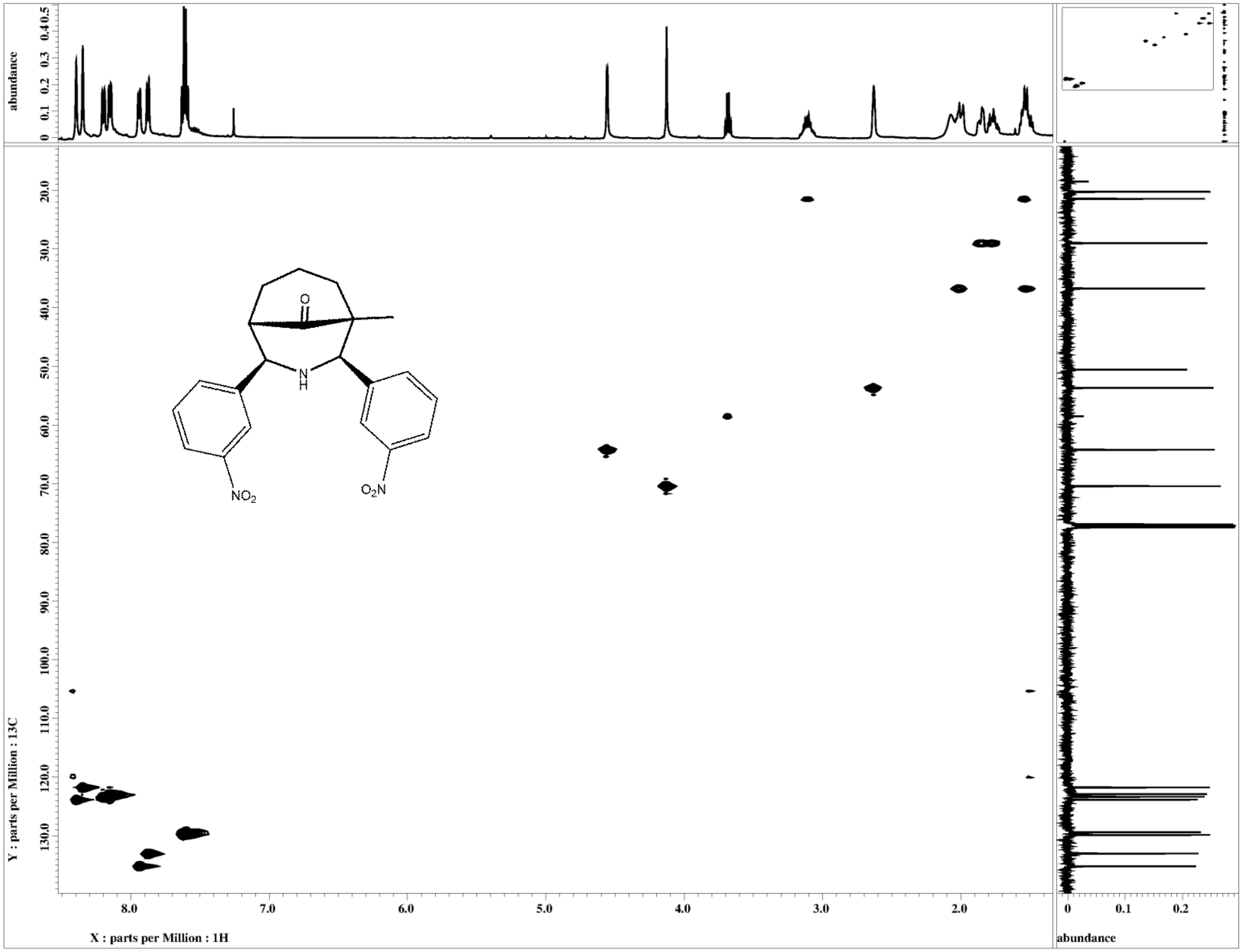

HSQC spectrum of compound **7**

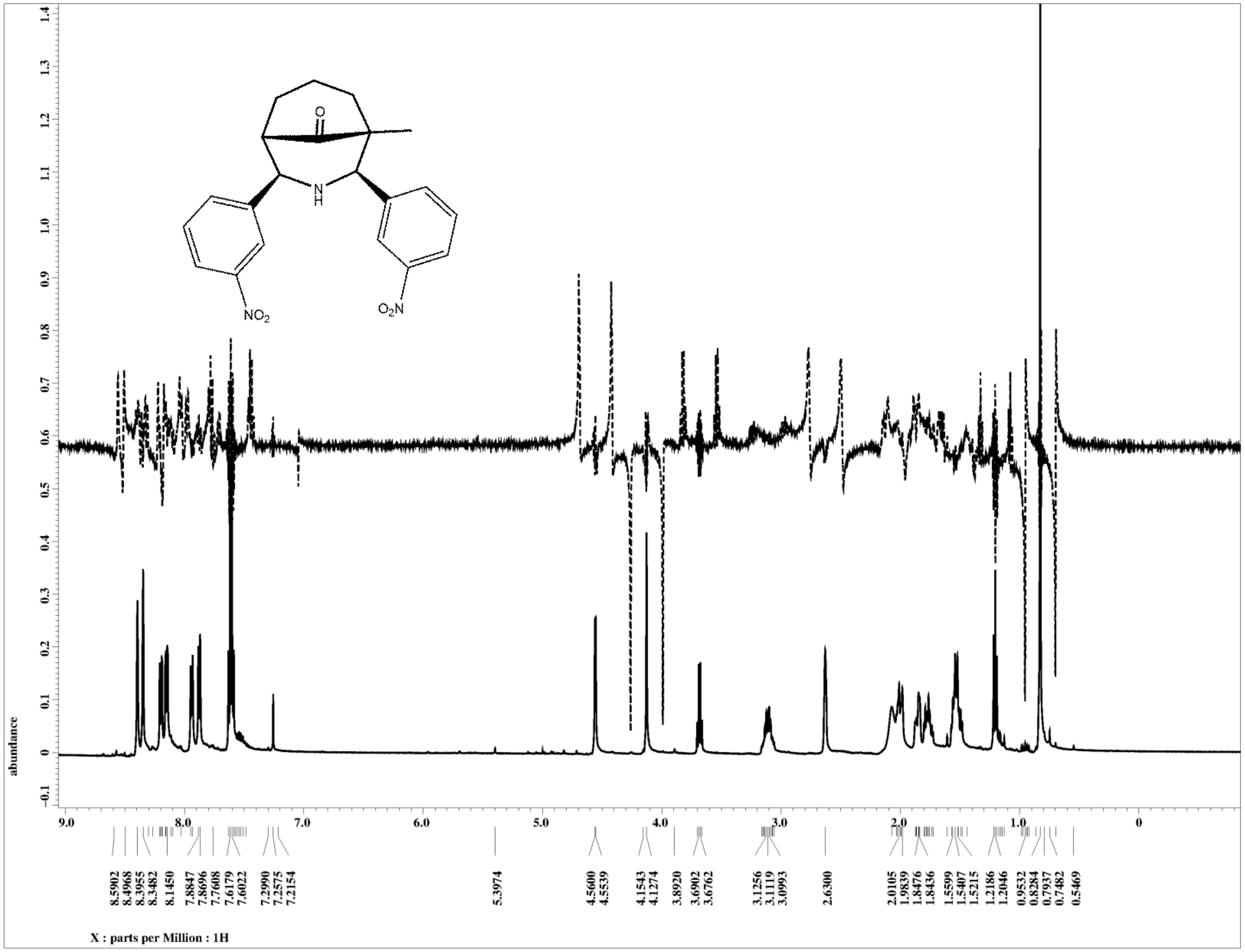

DQF spectrum of compound **7**
